# A Sequential Generalized Nonparametric Classification Method for Small-Scale Cognitive Diagnostic Assessment

**DOI:** 10.3390/bs16040528

**Published:** 2026-04-01

**Authors:** Junjie Li, Huijing Zheng, Chunhua Kang, Yan Cai, Dongbo Tu

**Affiliations:** 1 School of Psychology, Jiangxi Normal University, Nanchang 330022, China; ajie202310@163.com (J.L.);; 2 Teacher Development Center of Yongjia County, Wenzhou 325199, China; zhj123@zjnu.edu.cn; 3 School of Psychology, Zhejiang Normal University, Jinhua 321004, China; akang@zjnu.cn

**Keywords:** cognitive diagnosis, polytomous response data, nonparametric classification, small sample assessment

## Abstract

Small-scale (e.g., classroom) assessment represents the most common and needed scenario for cognitive diagnostic testing. In such settings, polytomously scored items (e.g., constructed-response tasks) are widely used, as they provide more fine-grained measurement of students’ skills and cognitive processes. However, a significant gap remains between the current methods and pressing practical needs. On one hand, parametric cognitive diagnosis models capable of handling polytomous response data require large samples for stable estimation, making them unsuitable for small-scale classroom use. On the other hand, existing nonparametric classification methods, while robust in small samples, are largely confined to dichotomous (0/1) response data. There is a lack of dedicated nonparametric methods for polytomous responses, creating a disconnect between practical testing and diagnostic tools. To address this real-world necessity, this study proposes the seq-GNPED method. It extends the generalized nonparametric classification framework to polytomous data by introducing weighted ideal category response and a collapsed class iterative algorithm. Simulations and empirical applications confirm that seq-GNPED achieves robust and accurate diagnosis under small sample conditions where parametric models falter, effectively leveraging the informational richness of polytomous items. This work bridges a critical gap by providing a practical, nonparametric tool tailored for fine-grained, classroom-ready cognitive diagnosis.

## 1. Introduction

**Figure 1:**
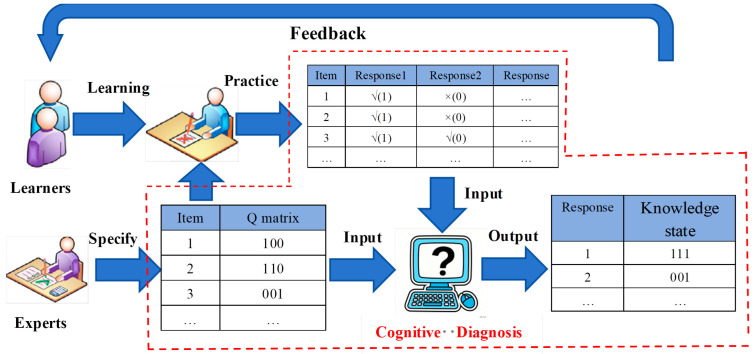
Schematic of cognitive diagnosis.

Cognitive Diagnostic Assessment (CDA) represents a significant advancement in modern educational and psychological measurement. The core goal of CDA is to provide in-depth diagnostic information to support precise instructional intervention (de la Torre et al., 2018; Z. Tan et al., 2023; Tang & Zhan, 2021). It goes beyond the limitation of traditional tests that offer only a single ability score, enabling detailed diagnosis of an individual’s mastery status on fine-grained cognitive skills or knowledge structures (collectively referred to as “attributes”). By mapping examinees’ item responses to attributes predefined by a *Q*-matrix, CDA can generate a refined “cognitive profile,” which essentially represents the knowledge state, typically expressed as a binary vector indicating the mastery of each attribute (Figure 1). This process provides empirical evidence for personalized instruction and targeted intervention, strongly promoting the practice of “assessment for learning”. Therefore, CDA has become a key component of intelligent teaching systems (ITS, Su et al., 2022; Qi et al., 2022; Huang et al., 2024). Its diagnostic information holds significant value for formative classroom assessment and has gained increasingly wide practical application (Su et al., 2022; Qi et al., 2022; Huang et al., 2024; Yang et al., 2022; X. Chen et al., 2024).

Effective cognitive diagnosis requires rigorous psychometric model support. Existing methods mainly fall into two categories: parametric cognitive diagnosis models and nonparametric cognitive diagnosis methods. Parametric models such as the deterministic input noisy “AND” gate model (DINA, Haertel, 1989), the deterministic inputs, noisy ‘OR’ gate model (DINO; J. L. Templin & Henson, 2006), reduced reparametrized unified model (R-RUM; Hartz, 2002), and especially the generalized diagnostic model (G-DINA), are built upon explicit probability distribution assumptions. They quantify the relationship between attribute mastery and the probability of a correct response by estimating item parameters (de la Torre, 2011; Henson et al., 2009). The advantage of such models lies in their well-established statistical framework. When correctly specified and with sufficient sample size, they can provide rich item quality information and high diagnostic accuracy. However, their application also has clear limitations: first, parameter estimation typically requires large samples to ensure stability and accuracy; second, the complex processes of model identification, estimation, and evaluation place high professional demands on users (C. Y. Chiu & Douglas, 2013; C.-Y. Chiu et al., 2018). C.-Y. Chiu et al. (2018) noted that in classroom-sized samples (*N* = 30–50), the stability of parameter estimation for 0–1 data using parametric models decreases significantly, with classification accuracy falling more than 20% below that of nonparametric methods. The limitations of parametric models in small sample contexts have been well documented in existing empirical studies. For instance, C.-Y. Chiu et al. (2018) compared the Generalized Nonparametric Classification (GNPC) method with the parametric G-DINA method using data from the Fraction Addition and Subtraction Test (FAST). This dataset consisted of 18 small-scale classes (ranging from 7 to 30 students per class), perfectly representing classroom assessment scenarios. The results revealed that when sample sizes were small, the G-DINA method failed to produce reasonable parameter estimates: most item parameter estimates were either 0 or 1, indicating estimation failure. More importantly, in terms of classification accuracy, the nonparametric GNPC method outperformed the G-DINA method in all 18 classes, correctly classifying an average of 24.5% more students, with advantages reaching as high as 51.9% in some classes. These empirical findings provide strong evidence for the serious challenges faced by parametric models in small-scale classroom assessment settings and directly support the value of nonparametric methods in such contexts.

In contrast, nonparametric classification methods rely on weaker statistical assumptions (C. Y. Chiu & Douglas, 2013; C.-Y. Chiu et al., 2018; C. Y. Chiu & Köhn, 2019; C. Y. Chiu & Chang, 2021). These methods typically classify examinees directly into predefined attribute mastery patterns by computing the similarity or distance between observed response patterns and theoretical ideal response patterns. Their prominent advantages are: less stringent sample size requirements, robust performance under small sample conditions, and relatively simple computation. However, traditional nonparametric methods are primarily limited to handling dichotomously scored (0/1) data.

To better meet diverse real-world testing needs, both parametric and nonparametric methods have evolved along a path from unsaturated to saturated (or generalized) models. Unsaturated models (e.g., the DINA model or DINO model in the parametric framework) impose strong constraints on the item response function, assuming attributes interact in a specific, fixed manner (e.g., strictly compensatory or compensatory). This may lead to model misfit due to oversimplification when dealing with complex real data. To better fit diverse empirical data, saturated models have emerged, such as the generalized DINA (G-DINA; de la Torre, 2011) model, the log-linear CDM (LCDM; Henson et al., 2009) and the general diagnostic model (GDM; von Davier, 2008). In the parametric framework, saturated models like G-DINA employ fully parameterized item response functions. They allow each item to have a unique pattern of attribute effects, including all possible main effects and interactions, thereby flexibly accommodating complex cognitive mechanisms underlying different items. This flexibility makes saturated models more robust in practice. Correspondingly, generalized nonparametric classification methods (GNPC, C.-Y. Chiu et al., 2018) have also appeared in the nonparametric domain. Such methods handle complex item–attribute relationships by constructing weighted ideal responses for different attribute mastery patterns on specific items.

Despite these developments, a significant gap remains between the current methods and pressing practical needs. The primary issue concerns the type of data scoring. In actual educational and psychological tests, polytomously scored items (e.g., constructed-response items) are ubiquitous. They can depict examinees’ ability or trait levels more finely and continuously than dichotomous response data. Compared to traditional dichotomous items, polytomous items can provide richer and more valuable information with fewer items while maintaining equivalent measurement precision (Ma & de la Torre, 2016; Gao et al., 2020; Q. Tan et al., 2024). The polytomous item can effectively assess students’ knowledge mastery, skill levels, and psychological traits in complex problem-solving (Birenbaum & Tatsuoka, 1987; Birenbaum et al., 1992). More importantly, it can accurately reflect students’ cognitive processes and solution strategies, fully presenting their abilities to analyze, integrate, and apply knowledge (Kuo et al., 2016). In the past, researchers have developed a series of parametric cognitive diagnosis models capable of handling polytomous responses (Sun et al., 2013; Ma & de la Torre, 2016), such as the partial credit DINA model (PC-DINA; de la Torre, 2010), the graded response GDM model (pGDM; von Davier, 2008), the nominal response diagnostic model (NRDM; J. L. Templin et al., 2008), and the polytomous LCDM model (Hansen, 2013). Among these, the sequential G-DINA model developed by Ma and de la Torre (2016) is a commonly used saturated cognitive diagnosis model for polytomous response data. Although these parametric methods can handle polytomous response data, their parameter estimation typically requires large samples to ensure measurement precision, a condition often difficult to meet in practice. This is because polytomous cognitive diagnosis models generally use MMLE/EM, MCMC, and other methods that require large samples to achieve high estimation accuracy (C. Y. Chiu & Douglas, 2013; C.-Y. Chiu et al., 2018; C. Y. Chiu & Köhn, 2019).

An even more challenging reality is that classroom formative assessment, where diagnostic needs are most urgent and applications most widespread, often occurs under small sample conditions (dozens of examinees). In this context, parametric methods often face estimation difficulties and excessively large standard errors due to insufficient samples, limiting their practicality. Although nonparametric methods are naturally suited for small samples, existing generalized nonparametric classification (GNPC) methods are primarily designed for dichotomous response data. For example, the GNPC method proposed by C.-Y. Chiu et al. (2018) can relax the assumptions of constrained models and effectively handle situations where students master only part of the required knowledge but may still answer correctly. However, it should be noted that the GNPC method is not suitable for polytomous response data. Overall, although nonparametric cognitive diagnosis methods are suitable for small-scale assessments, they mainly focus on dichotomous response data and lack methods applicable to polytomously scored items. If polytomous response data change to dichotomous response data, a significant amount of information is lost, inevitably leading to reduced diagnostic accuracy (Lee et al., 2011; Ma & de la Torre, 2016; J. L. Templin & Henson, 2006). Therefore, developing a generalized method that can directly handle polytomous response data within a nonparametric framework and capture complex item–attribute relationships has become a necessary choice to meet the needs of real-world small sample testing scenarios (e.g., classroom assessment).

Given this, the present study aims to fill this critical method gap by proposing a generalized nonparametric cognitive diagnosis method for polytomously scored data. The specific objective is to construct a classification method within the nonparametric framework that can handle polytomous response data and complex item–attribute relationships. The outcomes of this research are expected to provide front-line educators with a robust, reliable, and easy-to-operate diagnostic tool under realistic constraints (small samples, polytomous responses), effectively promoting the transition of cognitive diagnosis from theoretical models to large-scale, educational practice, ultimately serving the ultimate goal of enhancing personalized learning outcomes.

## 2. Background

### 2.1. Generalized Nonparametric Classification (GNPC) Method

The generalized nonparametric classification (GNPC) method is an important nonparametric technique developed in the field of cognitive diagnosis to address small sample scenarios (C.-Y. Chiu et al., 2018; C. Y. Chiu & Köhn, 2019; C. Y. Chiu & Chang, 2021). Its core goal is to achieve robust and accurate classification of examinees’ attribute mastery patterns without presupposing a specific form of cognitive diagnosis model (CDM). Compared to traditional nonparametric methods, GNPC significantly enhances flexibility by introducing an adaptive weighting mechanism that accommodates different cognitive processing assumptions (e.g., conjunctive, compensatory), thereby approaching the flexibility of parametric saturated models (e.g., G-DINA) while retaining the core advantages of nonparametric methods: low sample size requirements and computational simplicity.

The cornerstone of the GNPC method is the concept of weighted ideal responses. While traditional nonparametric methods rely on a single ideal response pattern (typically conjunctive or disjunctive), GNPC integrates two fundamental ideal response patterns (conjunctive and disjunctive) through a data-driven weight.

For item j and a specific attribute mastery class l, the weighted ideal response $\eta_{\mathrm{lj}}^{\left(w\right)}$ is defined as:

(1) $$\eta_{\mathrm{lj}}^{\left(w\right)}\;={\;w}_{\mathrm{lj}}\eta_{\mathrm{lj}}^{\left(c\right)}\;+\;\left(1\;-\;w_{\mathrm{lj}}\right)\eta_{\mathrm{lj}}^{\left(d\right)},$$

where $\eta_{\mathrm{lj}}^{\left(c\right)}\;=\;\prod_{k=1}^{K_{j}^{*}}\alpha_{\mathrm{lk}}^{q_{\mathrm{jk}}}$ is the conjunctive ideal response. It assumes that an examinee must master all attributes required by item j ($q_{\mathrm{jk}}\;=\;1$) to answer correctly, reflecting a non-compensatory mechanism similar to the DINA model. $\eta_{\mathrm{lj}}^{\left(d\right)}\;=\;1-\prod_{k=1}^{K_{j}^{*}}{\left(1-\alpha_{\mathrm{lk}}\right)}^{q_{\mathrm{jk}}}$ is the disjunctive ideal response. It assumes that an examinee who masters at least one required attribute has the possibility to answer correctly, reflecting a compensatory mechanism similar to the DINO model. $w_{\mathrm{lj}}\;\in \;\left[0,1\right]$ is the key adaptive weight parameter. It is estimated from the data and determines the relative contribution of conjunctive and disjunctive assumptions for a specific item and examinee class. When $w_{\mathrm{lj}}\;=\;1$, the model fully follows the conjunctive assumption; when $w_{\mathrm{lj}}\;=\;0$, it fully follows the disjunctive assumption; and intermediate values of $w_{\mathrm{lj}}$ represent a mixed or partially compensatory cognitive process.

#### Implementation Steps of the GNPC Method

**Step 1: Data grouping**

Based on the *Q*-matrix, all examinees are grouped according to their reduced attribute mastery pattern $\alpha_{l}^{*}$ for item j (i.e., containing only attributes examined by item j), forming different classes $C_{l}$.

**Step 2: Compute difference between observed and ideal responses**

For each item j and each class $C_{l}$, compute the sum of squared differences between the observed scores $y_{\mathrm{ij}}$ of all examinees in the class and the weighted ideal response $\eta_{\mathrm{lj}}^{\left(w\right)}$:

(2) $$d_{\mathrm{lj}}\;=\;\sum_{i\in C_{l}}{\left(y_{\mathrm{ij}}-\eta_{\mathrm{lj}}^{\left(w\right)}\right)}^{2}=\sum_{i\in C_{l}}{\left(y_{\mathrm{ij}}-\left[w_{\mathrm{lj}}\eta_{\mathrm{lj}}^{\left(c\right)}+\left(1-w_{\mathrm{lj}}\right)\eta_{\mathrm{lj}}^{\left(d\right)}\right]\right)}^{2}.$$

**Step 3: Estimate weight parameter**

The optimal weight ${\hat{w}}_{\mathrm{lj}}$ is estimated by minimizing the difference $d_{\mathrm{lj}}$. This optimization problem has a solution:

(3) $${\hat{w}}_{\mathrm{lj}}\;=\;\frac{\sum_{i\in C_{l}}\left(y_{\mathrm{ij}}-\eta_{\mathrm{lj}}^{\left(d\right)}\right)}{\left|C_{l}\right|\cdot \left(\eta_{\mathrm{lj}}^{\left(c\right)}-\eta_{\mathrm{lj}}^{\left(d\right)}\right)},$$

where $\left|C_{l}\right|$ is the number of examinees in class $C_{l}$. This estimator intuitively reflects the deviation of observed data from the pure disjunctive expectation, scaled by the inverse of the difference between conjunctive and disjunctive expectations.

**Step 4: Construct ideal response patterns and perform classification**

(1) **Pattern construction**: Using the estimated weights ${\hat{w}}_{\mathrm{lj}}$ for all items, compute the weighted ideal response scores on all J test items for each possible full attribute mastery pattern $\alpha_{m}$ (total $2^{K}$ patterns), thereby constructing the complete weighted ideal response vector ${\hat{\eta }}_{m}^{\left(w\right)}\;=\;\left({\hat{\eta }}_{m1}^{\left(w\right)},\;{\hat{\eta }}_{m2}^{\left(w\right)},\;\ldots ,{\hat{\;\eta }}_{\mathrm{mJ}}^{\left(w\right)}\right)$.

(2) **Distance calculation**: For a new examinee’s observed response vector $y_{i}\;=\;\left(y_{i1},{\;y}_{i2},\;\ldots ,{\;y}_{\mathrm{iJ}}\right)$, compute its distance to each weighted ideal response vector ${\hat{\eta }}_{m}^{\left(w\right)}$, typically using the squared Euclidean distance:

(4) $$d\left(y_{i},{\hat{\eta }}_{m}^{\left(w\right)}\right)\;=\;\sum_{j=1}^{J}{\left(y_{\mathrm{ij}}-{\hat{\eta }}_{\mathrm{mj}}^{\left(w\right)}\right)}^{2}$$

(3) **Classification decision**: Assign the examinee to the attribute mastery pattern corresponding to the ideal response pattern with the smallest distance to their observed response pattern:

(5) $${\hat{\alpha }}_{i}=\underset{m\in \{1,2,\ldots ,M\}}{\mathrm{argmin}}\;d\left(y_{i},{\hat{\eta }}_{m}^{\left(w\right)}\right).$$

The main contributions and advantages of the GNPC method are: (1) **Excellent model flexibility**: Through data-driven weights $w_{\mathrm{lj}}$, GNPC can adaptively capture complex item–attribute relationships ranging from pure conjunctive and pure disjunctive to various intermediate forms, without any prior model specification or selection. This makes its generality comparable to the parametric saturated model G-DINA. (2) **Strong robustness in small samples**: Its estimation algorithm is primarily based on counting and algebraic operations, avoiding the complex iterative estimation and large sample requirements of parametric models. Research confirms that under small sample conditions, GNPC’s classification accuracy is significantly better than parametric methods requiring precise parameter estimation. (3) **Computational and implementation simplicity**: The algorithm flow is clear, computationally efficient, and easy to implement in practice and programming.

The success of GNPC demonstrates the feasibility and great potential of constructing generalized diagnostic models within a nonparametric framework. However, as noted in the literature, GNPC and its derivative applications are mainly designed for dichotomously scored (0/1) data. This precisely highlights the core of the current research gap: how to creatively extend and apply the flexible and robust generalized nonparametric diagnostic philosophy represented by GNPC to polytomous response data containing richer information. This study is dedicated to this aim, seeking to develop a new generalized nonparametric cognitive diagnosis method that inherits all the advantages of GNPC (small-sample-friendly, model-flexible, computationally efficient) while directly handling polytomous responses, thereby meeting the urgent need for refined diagnosis in real-world small sample scenarios such as classroom assessment.

### 2.2. Seq-GDINA Model

The Sequential G-DINA (Seq-GDINA) model, proposed by Ma and de la Torre (2016), is a saturated cognitive diagnosis model suitable for polytomously scored data. Unlike traditional cognitive diagnosis models that only handle dichotomous response data, Seq-GDINA directly links attribute mastery to item scoring categories, achieving flexible modeling of complex cognitive processes by constructing a category-level *Q*-matrix (*Q_c_* matrix).

Assume a polytomous item involves several sequentially executed cognitive categories, with different score categories corresponding to the completion of different categories. Let item j have $H_{j}\;+\;1$ score categories ($0,\;1,\;\ldots ,{\;H}_{j}$), where category h indicates the examinee successfully completed the first h categories. Each category may involve different attribute sets.

***Q_c_* Matrix**: The traditional item–attribute association *Q*-matrix is extended to a *Q_c_* matrix with dimensions $\sum_{j=1}^{J}H_{j}\;\times \;K$, where each row corresponds to a score category, listing the attributes required for a correct response in that category. It explicitly specifies the attribute set required for each category, typically assuming sequential dependency between categories.

#### 2.2.1. Category Processing Function

Define $S_{j}\left(h|\alpha_{c}\right)$ as the probability that an examinee with attribute pattern $\alpha_{c}$ answers the hth category correctly given successful completion of the first h − 1 category. This function is parameterized using the G-DINA framework:

(6) $$S_{j}\left(h|\alpha_{jh}^{*}\right)\;=\;\phi_{jh0}+\sum_{k=1}^{K_{jh}^{*}}\phi_{jhk}\alpha_{\mathrm{lk}}+\sum_{k’=k+1}^{K_{jh}^{*}}\sum_{k=1}^{K_{jh}^{*}-1}\phi_{jhkk’}\alpha_{\mathrm{lk}}\alpha_{\mathrm{lk}’}\;+\;\cdots \;+\;\phi_{jh12\ldots K_{jh}^{*}}\prod_{k=1}^{K_{jh}^{*}}\alpha_{\mathrm{lk}},$$

where $\alpha_{jh}^{*}$ is the reduced attribute vector corresponding to category h; $\phi_{jh0}$ is the intercept, representing the baseline probability of correctness when no required attributes are mastered; $\phi_{jhk}$ is the main effect of attribute k; and $\phi_{jhkk}$, and higher-order terms represent interaction effects between attributes.

#### 2.2.2. Category Response Probability

The probability that an examinee scores h on item j is:

(7) $$P\left(X_{j}\;=\;h|\alpha_{c}\right)\;=\;\left[1-S_{j}\left(h\;+\;1|\alpha_{c}\right)\right]\prod_{x=0}^{h}S_{j}\left(x|\alpha_{c}\right),$$

with the conventions $S_{j}\left(0|\alpha_{c}\right)\;=\;1$ and $S_{j}\left(H_{j}\;+\;1|\alpha_{c}\right)\;=\;0$.

By explicitly modeling category–attribute relationships, Seq-GDINA can flexibly depict the cognitive mechanisms behind different score categories, making it particularly suitable for constructed-response items with obvious sequential categories. Its saturated form includes main and interaction effects, providing strong data-fitting capability.

However, as a parametric method, Seq-GDINA still relies on relatively large samples to ensure parameter estimation stability, limiting its application in small-scale classroom assessment. Therefore, developing nonparametric classification methods suitable for polytomous response data becomes an important direction for promoting the adoption of cognitive diagnosis in everyday teaching practice.

## 3. Sequential Generalized Nonparametric Classification Method with Euclidean Distance (seq-GNPED)

### 3.1. seq-GNPED Algorithm

Assume a test consists of J items, each employing polytomous responses, with the maximum score for item j being $S_{j}$ (e.g., $0,\;1,\;\ldots ,{\;S}_{j}$), measuring K attributes. To handle polytomous response data, a categorywise scoring mechanism is introduced: each polytomous item is decomposed into $S_{j}$ categories, with each category corresponding to a dichotomous score (0/1). The *Q_c_* matrix (Ma & de la Torre, 2016) describes the attribute set examined at each category. For item j, the *Q_c_* vector for category h is denoted $q_{\mathrm{jh}}\;=\;\left(q_{\mathrm{jh}1},\;\ldots ,\;q_{\mathrm{jhK}}\right)$, where $q_{\mathrm{jhk}}\;=\;1$ indicates category h measures attribute k, and 0 otherwise.

The actual polytomous response $X_{\mathrm{ij}}\in \{0,\;1,\;\ldots ,{\;S}_{j}\}$ is transformed into a category response vector $Y_{\mathrm{ij}}\;=\;\left(y_{\mathrm{ij}1},\ldots ,y_{\mathrm{ij}S_{j}}\right)$ as follows:

(8) $$y_{\mathrm{ijh}}\;=\;\left\{\begin{array}{ll} 1, & \mathrm{if}{\;X}_{\mathrm{ij}}\;\geq \;h\;(\mathrm{i.e.,}\;\mathrm{examinee}\;i\;\mathrm{correctly}\;\mathrm{completed}\;\mathrm{up}\;\mathrm{to}\;\mathrm{category}\;h\;\mathrm{in}\;\mathrm{item}\;j),\\ 0, & \mathrm{if}{\;X}_{\mathrm{ij}}\;<\;h\;(\mathrm{i.e.,}\;\mathrm{failed}\;\mathrm{to}\;\mathrm{correctly}\;\mathrm{complete}\;\mathrm{up}\;\mathrm{to}\;\mathrm{category}\;h). \end{array}\right.$$

#### 3.1.1. Construction of Ideal Category Response

For each attribute mastery pattern $\alpha_{m}\;=\;\left(\alpha_{1},\;\ldots ,{\;\alpha }_{M}\right)$, $\eta_{\mathrm{mjh}}^{\left(c\right)}$ and $\eta_{\mathrm{mjh}}^{\left(d\right)}$ respectively denote its conjunctive and disjunctive ideal category response on category h of item j. Consider two baseline models:

**Non-compensatory baseline (DINA model,** Haertel, 1989**)**:

The ideal response for category b (b = 1, …, h) (i.e., whether all required attributes for that category are mastered) is defined as:

(9) $$\eta_{\mathrm{mjb}}^{\left(c\right)}\;=\;\prod_{k=1}^{K}\alpha_{\mathrm{mk}}^{q_{\mathrm{jbk}}}.$$

Then, the conjunctive ideal category response for completing up to category h is:

(10) $$\eta_{\mathrm{mjh}}^{\left(c\right)}\;=\;\prod_{b=1}^{h}\eta_{\mathrm{mjb}}^{\left(c\right)}.$$

This definition embodies conjunctive logic: one must master all required attributes from the first category to the *h*th category to score 1.

**Fully Compensatory Baseline (DINO model,** J. L. Templin & Henson, 2006**)**:

The ideal response for category b (i.e., whether at least one required attribute for that category is mastered) is defined as:

(11) $$\eta_{\mathrm{mjb}}^{\left(d\right)}\;=\;1-\prod_{k=1}^{K}{\left(1-\alpha_{\mathrm{mk}}\right)}^{q_{\mathrm{jbk}}}.$$

Then, the disjunctive ideal category response for completing up to category h is:

(12) $$\eta_{\mathrm{mjh}}^{\left(d\right)}\;=\;\prod_{b=1}^{h}\eta_{\mathrm{mjb}}^{\left(d\right)}.$$

This definition embodies disjunctive logic: score 1 means that one masters at least one required attribute in each category from the first category to the *h*th category.

To accommodate more general item response mechanisms (e.g., the saturated seq-GDINA model), a weighted ideal category response is introduced:

(13) $$\eta_{\mathrm{mjh}}^{\left(w\right)}\;=\;w_{\mathrm{lj}h}\;\eta_{\mathrm{mjh}}^{\left(c\right)}\;+\;\left(1-w_{\mathrm{ljh}}\right)\;\eta_{\mathrm{mjh}}^{\left(d\right)},$$

where $w_{\mathrm{lj}h}\;\in \;\left[0,1\right]$ is a weight, and l denotes a collapsed attribute class, defined as follows.

#### 3.1.2. Collapsed Attribute Class

For category h of item j, only the attributes corresponding to non-zero elements in its *Q_c_* vector $q_{\mathrm{jh}}$ are actually examined in this category. Let the number of such attributes be $K_{\mathrm{jh}}^{*}\;\leq \;K$. Rearrange these attributes to the first $K_{\mathrm{jh}}^{*}$ positions, obtaining a reduced *Q_c_* vector $q_{\mathrm{jh}}^{*}$. Among the original $2^{K}$ attribute patterns, only the first $K_{jh}^{*}$ attributes affect the ideal response for this category; the remaining $K-K_{\mathrm{jh}}^{*}$ attributes have no effect. Therefore, all patterns with identical values on the first $K_{\mathrm{jh}}^{*}$ attributes are indistinguishable on this category and are “collapsed” into the same class, called a collapsed attribute class, denoted $C_{l}$, where $l\;=\;1,\;\ldots ,\;2^{K_{\mathrm{jh}}^{*}}$.

**Example**: Suppose K = 3, and the *Q_c_* vector for category h of item j is $q_{\mathrm{jh}}\;=\;\left(1,1,0\right)$, so $K_{\mathrm{jh}}^{*}\;=\;2$ and $q_{\mathrm{jh}}^{*}\;$= (1,1). Attribute patterns (0,0,0) and (0,0,1) have the same first two positions (0,0) and are collapsed into class $C_{1}$; similarly, (1,0,0) and (1,0,1) are collapsed into $C_{2}$, etc.

In the weighted ideal response formula, the weight $w_{\mathrm{ljh}}$ is associated with the collapsed class l, reflecting the degree to which examinees in that class tend toward a conjunctive or disjunctive response mechanism on category h of item j.

#### 3.1.3. Weight Parameters Estimation

For each collapsed class l, the weight $w_{\mathrm{ljh}}$ is estimated by:

(14) $${\hat{w}}_{\mathrm{ljh}}\;=\;\frac{\sum_{i\in C_{l}}\left(y_{\mathrm{ijh}}-\eta_{\mathrm{ljh}}^{\left(d\right)}\right)}{\left|C_{l}\right|\cdot \left(\eta_{\mathrm{ljh}}^{\left(c\right)}-\eta_{\mathrm{ljh}}^{\left(d\right)}\right)},$$

where $C_{l}$ is the set of examinees belonging to collapsed class l, and $\left|C_{l}\right|$ is its size. Where $y_{\mathrm{ijh}}$ is the category response of examinee *i* about category h of item j. Here, $\eta_{\mathrm{ljh}}^{\left(c\right)}$ and $\eta_{\mathrm{ljh}}^{\left(d\right)}$ denote the conjunction and disjunction category response, respectively, of the collapsed class l for the category h of item j.

#### 3.1.4. Classification Rule

The attribute pattern $\alpha_{i}$ for examinee i is determined by minimizing the Euclidean distance between their actual category response vector and the weighted ideal category response vector for each candidate pattern:

(15) $${\hat{\alpha }}_{i}\;=\;\underset{m=1,\ldots ,2^{K}}{\mathrm{argmin}}\sqrt{\sum_{j=1}^{J}\sum_{h=1}^{S_{j}}{\left(y_{\mathrm{ijh}}-\eta_{\mathrm{mjh}}^{\left(w\right)}\right)}^{2}}.$$

#### 3.1.5. Iterative Algorithm Flow

**Figure 2:**
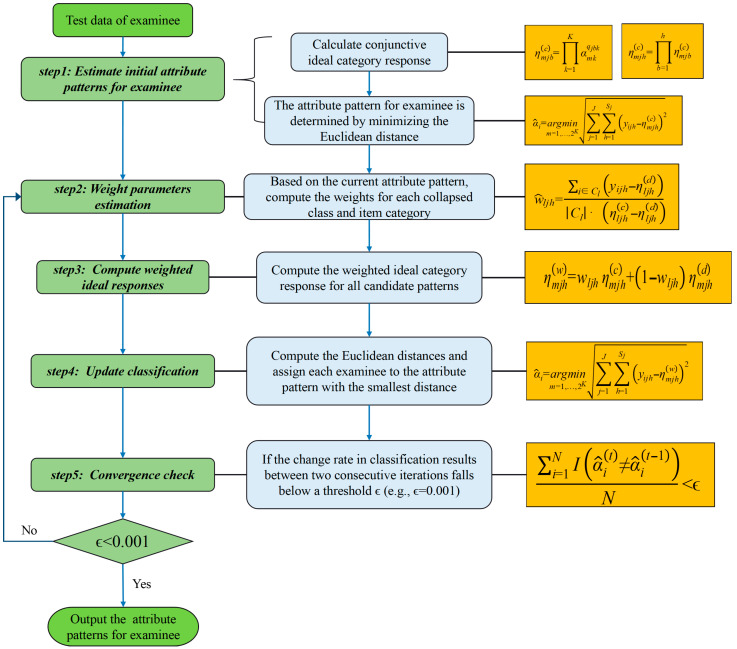
Flowchart of seq-GNPED algorithm.

The seq-GNPED uses the following iterative algorithm for parameter estimation and classification (Figure 2):

(1) **Initialization**: Use the equation ${\hat{\alpha }}_{i}\;=\;\underset{m=1,\ldots ,2^{K}}{\mathrm{argmin}}\sqrt{\sum_{j=1}^{J}\sum_{h=1}^{S_{j}}{\left(y_{\mathrm{ijh}}-\eta_{\mathrm{mjh}}^{\left(c\right)}\right)}^{2}}$, (i.e., $\eta_{\mathrm{mjh}}^{\left(c\right)}$ denote the conjunction category response of the attribute patterns m for the category h of item j) to estimate initial attribute patterns ${\hat{\alpha }}_{i}$ for examinee i.
(2) **Weight estimation**: Based on the current ${\hat{\alpha }}^{\left(t-1\right)}$, compute the weights ${\hat{w}}_{\mathrm{ljh}}^{\left(t\right)}$ for each collapsed class and item category using Equation (14).
(3) **Compute weighted ideal responses**: Compute the weighted ideal category response $\eta_{\mathrm{mjh}}^{\left(w\right)\left(t\right)}$ for all candidate patterns using Equation (13).
(4) **Update classification**: Compute the Euclidean distances using Equation (15) and assign each examinee to the attribute pattern with the smallest distance, obtaining ${\hat{\alpha }}^{\left(t\right)}$.
(5) **Convergence check**: If the change rate in classification results between two consecutive iterations falls below a threshold ϵ (e.g., ϵ = 0.001), i.e.,:
  (16) $$\frac{\sum_{i=1}^{N}I\left({\hat{\alpha }}_{i}^{\left(t\right)}\neq {\hat{\alpha }}_{i}^{\left(t-1\right)}\right)}{N}\;<\;\epsilon ,$$
  then stop iteration and output ${\hat{\alpha }}^{\left(t\right)}$; otherwise, set t ← t + 1 and return to Step 2.

### 3.2. Theoretical Rationale

Following the rationale of C.-Y. Chiu et al. (2018) for GNPC, we argue for the consistency of seq-GNPED from a statistical perspective.

**Assumption** **1.** *Consider a single item* j *category* h*; define* $\alpha^{\left(h\right)}$ *as the mastery vector of attributes examined in the first* h *categories (length* $K_{\mathrm{jh}}^{*}$*). We require the model to satisfy the following conventional psychometric assumption:*

(17) $$P\left(y_{\mathrm{ijh}}=1|\alpha^{\left(h\right)}=0\right)\;<\;0.5,$$

(18) $$P\left(y_{\mathrm{ijh}}=1|\alpha^{\left(h\right)}=1\right)\;>\;0.5,$$

*i.e., if none of the attributes examined in the first* h *categories are mastered, the probability of correctness is below chance level; if all are mastered, it is above chance level. This assumption ensures item discrimination.*

The likelihood function for examinee i is:

(19) $$L_{i}\;=\;\prod_{j=1}^{J}\prod_{h=1}^{S_{j}}P{\left(y_{\mathrm{ijh}}\;=\;1|\alpha_{i}\right)}^{y_{\mathrm{ijh}}}{\left[1-P\left(y_{\mathrm{ijh}}\;=\;1|\alpha_{i}\right)\right]}^{1-y_{\mathrm{ijh}}}.$$

The seq-GNPED classification rule seeks the attribute pattern $\alpha_{m}$ that minimizes the weighted Euclidean distance $D_{i}\;=\;\sqrt{\sum_{j,h}{\left(y_{\mathrm{ijh}}-\eta_{\mathrm{mjh}}^{\left(w\right)}\right)}^{2}}$. Below, we prove that, under the true saturated seq-GDINA model satisfying Assumption 1 and monotonicity (mastering more attributes does not decrease the probability of correctness), minimizing $D_{i}$ is equivalent to maximizing $L_{i}$.

**Proof.**  This study discuss three cases corresponding to the possible values of $\eta_{\mathrm{ljh}}^{\left(c\right)}$ and $\eta_{\mathrm{ljh}}^{\left(d\right)}$ in the collapsed class l.

Case 1: $\eta_{\mathrm{ljh}}^{\left(c\right)}\;=\;\eta_{\mathrm{ljh}}^{\left(d\right)}\;=\;0$

This implies that examinees in this class have mastered none of the attributes examined from the first category to the *h*th category of the item, i.e., $\alpha^{\left(h\right)}\;=\;0$. By Assumption 1:

(20) $$P_{\mathrm{jh}}\;:=\;P\left(Y_{\mathrm{ijh}}\;=\;1|\alpha^{\left(h\right)}\;=\;0\right)\;<\;0.5,$$

here, the weighted ideal category response $\eta_{\mathrm{mjh}}^{\left(w\right)\;}=\;0$ (since $\eta_{\mathrm{ljh}}^{\left(c\right)}\;=\;\eta_{\mathrm{ljh}}^{\left(d\right)}\;=\;0$).

Consider the two possibilities for examinee i’s actual response $y_{\mathrm{ij}h}$:

If $y_{\mathrm{ijh}}\;=\;0$, the Euclidean distance is $\sqrt{{\left(0\;-\;0\right)}^{2}}\;=\;0$, and the likelihood contribution for this category is $1-\;P_{\mathrm{jh}}$.

If $y_{\mathrm{ijh}}\;=\;1$, the Euclidean distance is $\sqrt{{\left(1\;-\;0\right)}^{2}}\;=\;1$, and the likelihood contribution is $P_{\mathrm{jh}}$.

Since $P_{\mathrm{jh}}\;<\;0.5$, we have $1\;-\;P_{\mathrm{jh}}\;>\;0.5\;>\;P_{\mathrm{jh}}$. Thus, when $y_{\mathrm{ijh}}\;=\;0$, not only is the Euclidean distance minimized (0), but the likelihood contribution is maximized ($1\;-{\;P}_{\mathrm{jh}}$). Therefore, in this case, minimizing Euclidean distance is equivalent to maximizing likelihood.

Case 2: $\eta_{\mathrm{ljh}}^{\left(c\right)}\;=\;\eta_{\mathrm{ljh}}^{\left(d\right)}\;=\;1$

This implies that examinees in this class have mastered all attributes examined from the first category to the *h*th category of the item, i.e., $\alpha^{\left(h\right)}\;=\;1$. By Assumption 1:

(21) $$P_{\mathrm{jh}}\;:=\;P\left(Y_{\mathrm{ijh}}\;=\;1|\alpha^{\left(h\right)}\;=\;1\right)\;>\;0.5,$$

here, $\eta_{\mathrm{mjh}}^{\left(w\right)}\;=\;1$ (since $\eta_{\mathrm{ljh}}^{\left(c\right)}\;=\;\eta_{\mathrm{ljh}}^{\left(d\right)}\;=\;1$).

Consider the two possibilities for examinee i’s actual response $y_{\mathrm{ijh}}$:

If $y_{\mathrm{ijh}}\;=\;1$, the Euclidean distance is $\sqrt{{\left(1\;-\;1\right)}^{2}}\;=\;0$, and the likelihood contribution is $P_{\mathrm{jh}}$.

If $y_{\mathrm{ijh}}\;=\;0$, the Euclidean distance is $\sqrt{{\left(0\;-\;1\right)}^{2}}\;=\;1$, and the likelihood contribution is $1\;-\;P_{\mathrm{jh}}$.

Since $P_{\mathrm{jh}}\;>\;0.5$, clearly $P_{\mathrm{jh}\;}>\;1\;-\;P_{\mathrm{jh}}$. Thus, when $y_{\mathrm{ijh}}\;=\;1$, the Euclidean distance is minimized (0) and the likelihood contribution is maximized ($P_{\mathrm{jh}}$). Therefore, in this case, minimizing Euclidean distance is equivalent to maximizing likelihood.

Case 3: $\eta_{\mathrm{ljh}}^{\left(d\right)}\;=\;1$ and $\eta_{\mathrm{ljh}}^{\left(c\right)}\;=\;0$

This implies that examinees in this class have mastered some but not all of the attributes examined from the first category to the *h*th category of the item. Suppose the number mastered is r ($0\;<\;r\;<\;K_{\mathrm{jh}}^{*}$), with corresponding correctness probability $P_{\mathrm{jh}}^{\left(r\right)}$. By monotonicity, $P_{\mathrm{jh}}^{\left(r\right)}\;\in \;\left(P_{\mathrm{jh}}^{\left(0\right)},P_{\mathrm{jh}}^{\left(K_{\mathrm{jh}}^{*}\right)}\right)$. The weighted ideal category response $\eta_{\mathrm{mjh}}^{\left(w\right)}\;=\;1\;-\;w_{\mathrm{ljh}}$.

From the weight estimation Formula (2), we have:

(22) $${\hat{w}}_{ljh}=1-\frac{\sum_{i\in C_{l}}y_{ijh}}{\left|C_{l}\right|},$$

when the sample size is sufficiently large, $\frac{\sum_{i\in C_{l}}y_{\mathrm{ijh}}}{\left|C_{l}\right|}$ converges in probability to $P_{\mathrm{jh}}^{\left(r\right)}$. Therefore, ${\hat{w}}_{\mathrm{ljh}}\;\to \;1\;-\;P_{\mathrm{jh}}^{\left(r\right)}$, and thus $\eta_{\mathrm{ljh}}^{\left(w\right)}\;\to \;P_{\mathrm{jh}}^{\left(r\right)}$.

Consider examinee i’s actual response $y_{\mathrm{ij}h}$:

If $y_{\mathrm{ij}h}\;=\;1$, the Euclidean distance converges to $\sqrt{{\left(1\;-{\;P}_{\mathrm{jh}}^{\left(r\right)}\right)}^{2}}$, and the likelihood contribution is $P_{\mathrm{jh}}^{\left(r\right)}$.

If $y_{\mathrm{ijh}}\;=\;0$, the Euclidean distance converges to $\sqrt{{\left(0\;-{\;P}_{\mathrm{jh}}^{\left(r\right)}\right)}^{2}}\;={\;P}_{\mathrm{jh}}^{\left(r\right)}$, and the likelihood contribution is $1\;-\;P_{\mathrm{jh}}^{\left(r\right)}$.

Thus, when $P_{\mathrm{jh}}^{\left(r\right)}\;>\;0.5$, we have $1\;-{\;P}_{\mathrm{jh}}^{\left(r\right)}\;<\;P_{\mathrm{jh}}^{\left(r\right)}$, so $y_{\mathrm{ijh}}\;=\;1$ corresponds to a smaller Euclidean distance and a larger likelihood contribution. Conversely, when $P_{\mathrm{jh}}^{\left(r\right)}\;<\;0.5$, we have $1\;-\;P_{\mathrm{jh}}^{\left(r\right)}\;>\;P_{\mathrm{jh}}^{\left(r\right)}$, so $y_{\mathrm{ijh}}=\;0$ corresponds to a smaller Euclidean distance and a larger likelihood contribution. Therefore, in this case, minimizing distance is still equivalent to maximizing likelihood.

Integrating the arguments across the three cases, for each category *h* of item *j*, minimizing the single-category Euclidean distance $\sqrt{{\left(y_{\mathrm{ijh}}\;-\;\eta_{\mathrm{mjh}}^{\left(w\right)}\right)}^{2}}$ is equivalent to maximizing the likelihood contribution of that category. Since the total likelihood $L_{i}$ is the product of category-wise likelihoods and the total distance $D_{i}$ is the sum of category-wise distances, minimizing $D_{i}$ is equivalent to maximizing $L_{i}$. This demonstrates that seq-GNPED possesses statistical consistency under conventional psychometric assumptions. **□**

## 4. Experimental Design and Results

### 4.1. Study 1: Data Generated from the seq-DINA Model

#### 4.1.1. Study Design

This study set five attributes and used a *Q_c_* matrix (see Table 1) consistent with that used in prior research (Ma & de la Torre, 2016). The total number of items was 21, including 16 polytomously scored items and 5 dichotomously scored items. The experimental design included four factors: (1) sample size (*N* = 30, 50, 100, 200, 300); (2) item quality (high, medium, low); (3) cognitive diagnosis method (seq-GDINA, seq-GNPED); and (4) distribution of examinee knowledge states (uniform, higher-order). Each experimental condition was replicated 100 times, and the average of the 100 experimental results was analyzed.

**Examinee knowledge states** were generated in two ways. Firstly, each attribute mastery pattern of uniform distribution had probability $1/2^{K}$. Secondly, the higher-order distribution adopts the model proposed by de la Torre and Douglas (2004). The probability that examinee *i* masters attribute *k* is given by:

(23) $$P(a_{k}=1|\theta_{i},\lambda_{k})=\frac{\exp\left(\lambda_{1k}(\theta_{i}-\lambda_{0k})\right)}{1+\exp\left(\lambda_{1k}(\theta_{i}-\lambda_{0k})\right)}$$

where $\theta_{i}$ denotes the latent trait of examinee *i*, generated from a standard normal distribution; $\lambda_{1k}$ represents the attribute discrimination parameter, simulated from a uniform distribution *U*(1,2); and $\lambda_{0k}$ represents the attribute difficulty parameter, generated equidistantly from the interval [−1.5, 1.5] (Ma & de la Torre, 2020a; Nájera et al., 2021).

The correct response probability $P_{j1}$ represents the probability of answering correctly when mastering all measured attributes, and $P_{j0}$ represents the probability when mastering none of the required attributes. Three levels of item quality are set: s ∈ {0.05, 0.10, 0.15}. For a sequentially scored item j, if the examinee has mastered all the attributes required from the first category to the *h*th category of the item, the probability $P_{j1}$ of scoring h is 1 − s, and the probability of scoring any other category (i.e., not h) is s. If the examinee has mastered none of the attributes required for the item, the probability $P_{j0}$ of scoring 0 is 1 − s, and the probability of scoring any other category (i.e., not 0) is s.

During simulation, the seq-DINA model from the R package GDINA was used to generate all parameters and response data (Ma & de la Torre, 2020b). Subsequently, the generated data were diagnosed using the two methods, seq-GDINA and seq-GNPED, to compare their diagnostic accuracy.

The Supporting Information can be downloaded at: https://osf.io/rfm3c/, accessed after 1 April 2026.

The evaluation metric was the Pattern Accuracy Ratio (PAR), calculated as:

(24) $$\mathrm{PAR}\;=\frac{1}{N}\sum_{i=1}^{N}I\left({\hat{\alpha }}_{i}\;=\;\alpha_{i}\right),$$

where the indicator function $I\left({\hat{\alpha }}_{i}\;=\;\alpha_{i}\right)$ takes the value 1 if examinee i’s estimated knowledge state ${\hat{\alpha }}_{i}$ matches the true state $\alpha_{i}$, and 0 otherwise. N is the total number of examinees. Higher *PAR* indicates more accurate estimation of overall knowledge states.

#### 4.1.2. Results

Table 2 presents the results of Simulation Study 1, comparing the Pattern Accuracy Ratio (PAR) between the seq-GNPED and seq-GDINA methods across various sample sizes, item quality levels (slipping parameters), and ability distributions.

**Figure 3:**
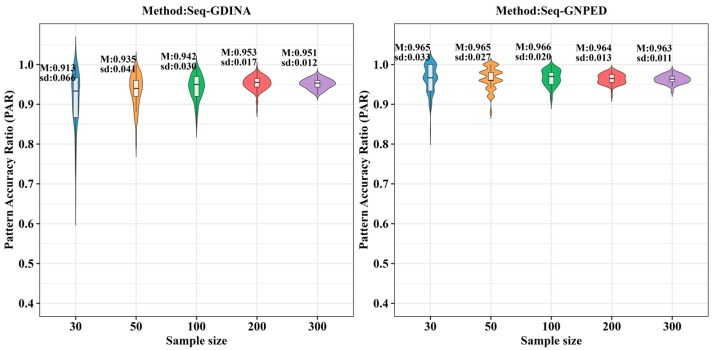
PAR of each method under the condition of uniform distribution with slipping probability 0.05.

**Figure 4:**
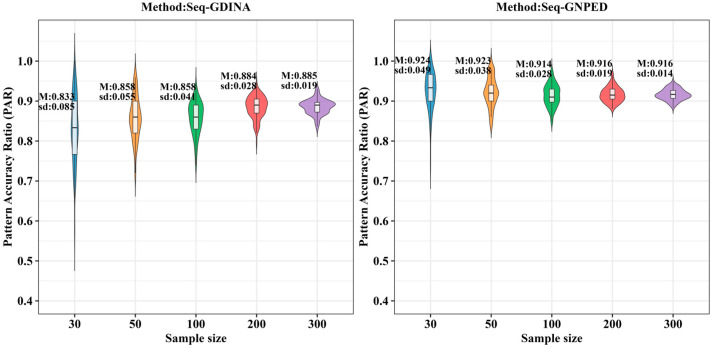
PAR of each method under the condition of uniform distribution with slipping probability 0.1.

**Figure 5:**
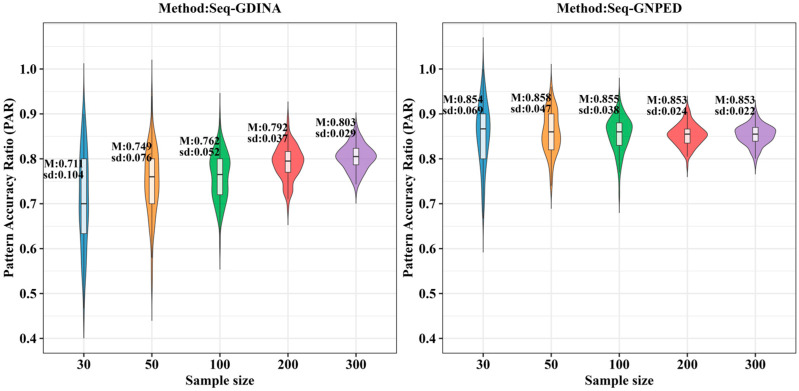
PAR of each method under the condition of uniform distribution with slipping probability 0.15.

**Figure 6:**
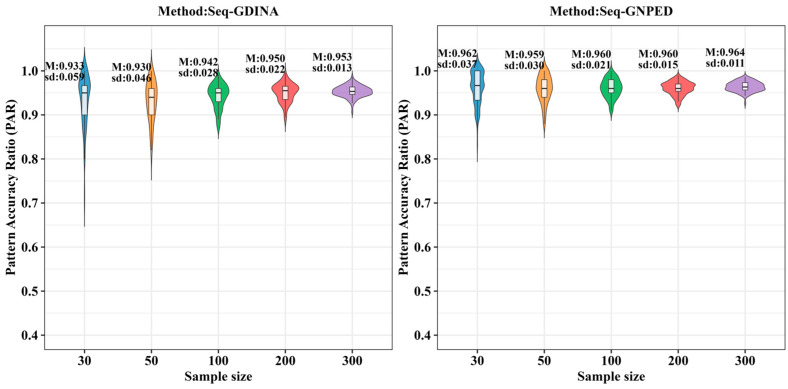
PAR of each method under the condition of higher-order distribution with slipping probability 0.05.

**Figure 7:**
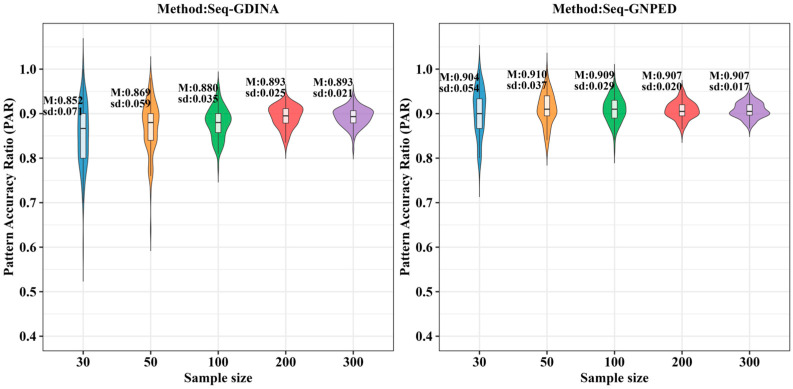
PAR of each method under the condition of higher-order distribution with slipping probability 0.1.

**Figure 8:**
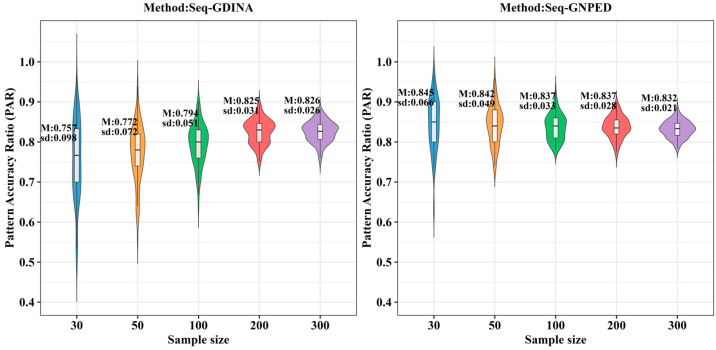
PAR of each method under the condition of higher-order distribution with slipping probability 0.15.

Figure 3, Figure 4, Figure 5, Figure 6, Figure 7 and Figure 8 show the PAR performance of each method under uniform and higher-order distributions, respectively. Under the uniform distribution, seq-GNPED’s PAR was higher than those of seq-GDINA. Under the item with slipping probability 0.15 condition, seq-GNPED’s PAR was significantly higher than those of seq-GDINA.

Specifically, the pattern recognition advantage of seq-GNPED over seq-GDINA became more pronounced as sample size decreased and item quality declined. Under high item quality (slipping probability 0.05), for sample sizes of 30, 50, and 100, seq-GNPED’s PAR exceeded seq-GDINA’s by 6%, 3%, and 3%, respectively. Under medium item quality (slipping probability 0.10), the corresponding advantages expanded to 9%, 6%, and 5%. Under low item quality (slipping probability 0.15), the advantages further increased to 14%, 11%, and 9%. These results indicate that the smaller the sample size, the more prominent seq-GNPED’s advantage in pattern classification accuracy, revealing the method’s feasibility and application value for small-scale classroom educational assessment, particularly in non-compensatory test situations. Under the higher-order distribution, the results were generally consistent with the trend under the uniform distribution.

### 4.2. Study 2: Data Generated from the seq-GDINA Model

#### 4.2.1. Study Design

This study design was identical to Study 1 in all experimental conditions (factor settings, item parameters, evaluation metrics, etc.), except that it included an additional condition: *Q*-matrix misspecification rate (0% and 10%) and used the parametric seq-GDINA model to generate all parameters and response data during the simulation data generation phase. The misspecified *Q*-matrix was constructed by randomly replacing the true *q*-vector with a *q*-vector from all candidate *q*-vectors (except for the *q*-vector of the item itself), while ensuring that a *q*-vector can have at most three misspecifications (Li & Chen, 2024). The GDINA model or the DINA model was randomly selected with a 50% probability as the link function for each item in the seq-GDINA model. The correct response probability $P_{j1}$ represents the probability of answering correctly when mastering all measured attributes, and $P_{j0}$ represents the probability when mastering none of the required attributes. For examinees with partial mastery patterns, their correct response probabilities were randomly generated from the interval $U\left(0.3,\;0.7\right)$.

#### 4.2.2. Results

**(1) Results of simulation study 2 with 0% *Q*-matrix misspecification rate**

Table 3 presents the results of Simulation Study 2 under the condition of 0% *Q*-matrix misspecification rate, comparing the Pattern Accuracy Ratio (PAR) between the seq-GNPED and seq-GDINA methods across various sample sizes, item quality levels (slipping parameters), and ability distributions.

**Figure 9:**
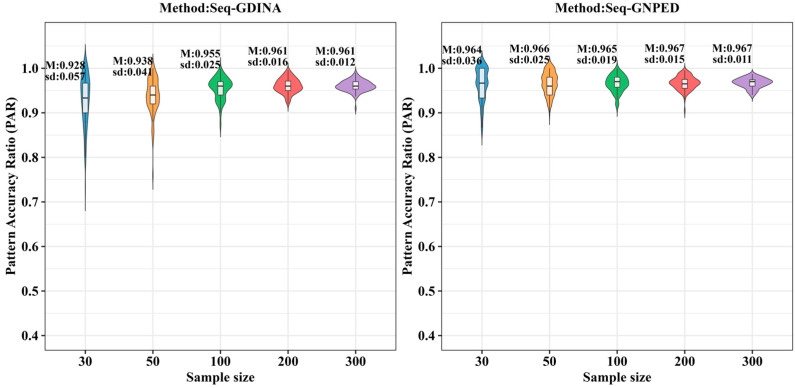
PAR of each method under the condition of uniform distribution with slipping probability 0.05 (0% *Q*-matrix misspecification rate).

**Figure 10:**
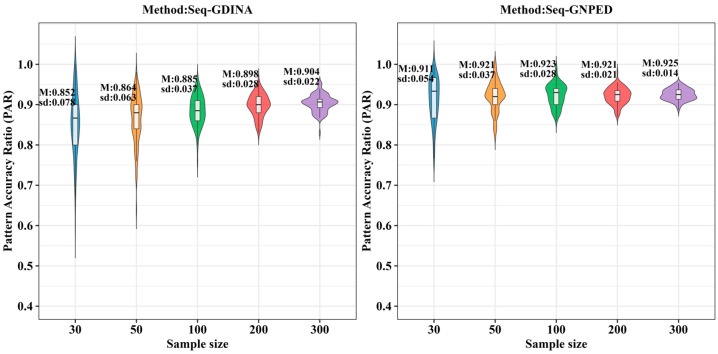
PAR of each method under the condition of uniform distribution with slipping probability 0.1 (0% *Q*-matrix misspecification rate).

**Figure 11:**
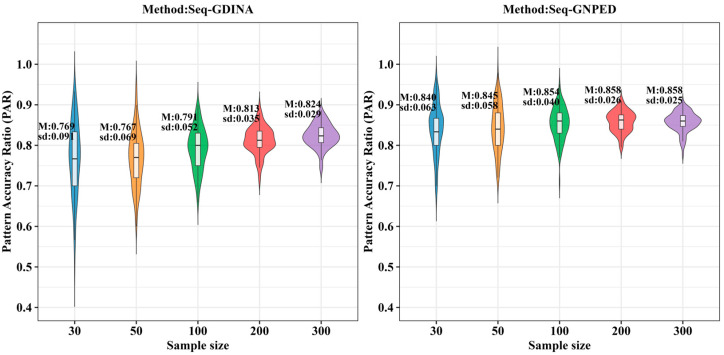
PAR of each method under the condition of uniform distribution with slipping probability 0.15 (0% *Q*-matrix misspecification rate).

**Figure 12:**
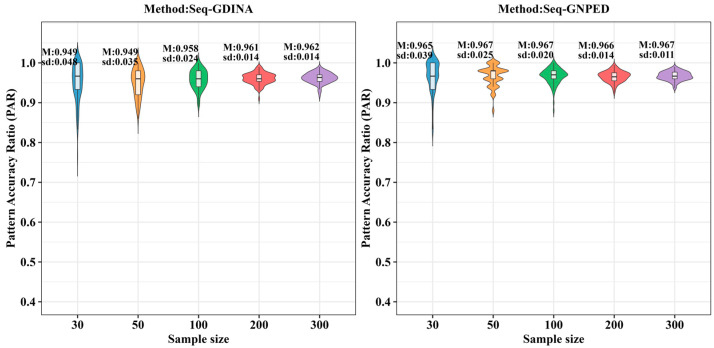
PAR of each method under the condition of higher-order distribution with slipping probability 0.05 (0% *Q*-matrix misspecification rate).

**Figure 13:**
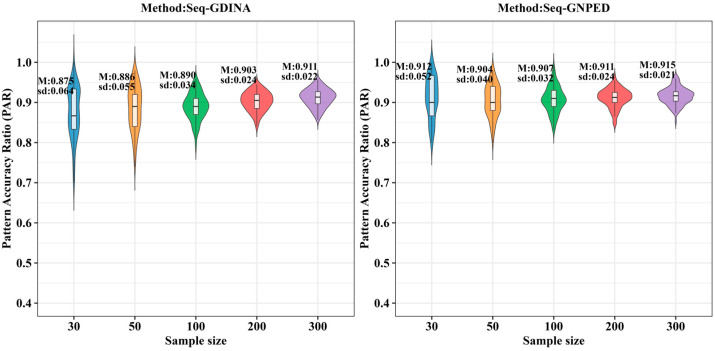
PAR of each method under the condition of higher-order distribution with slipping probability 0.1 (0% *Q*-matrix misspecification rate).

**Figure 14:**
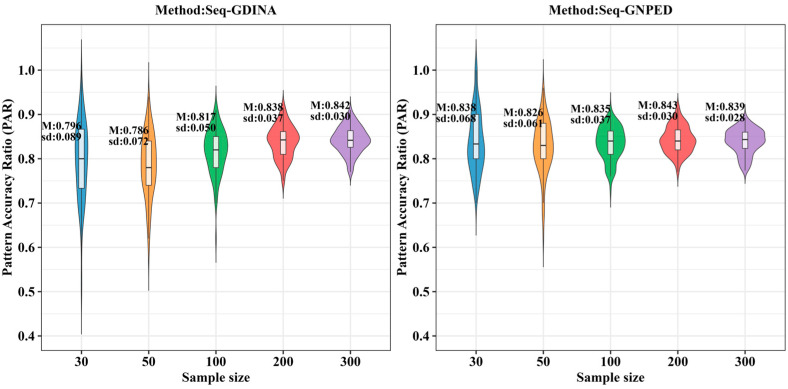
PAR of each method under the condition of higher-order distribution with slipping probability 0.15 (0% *Q*-matrix misspecification rate).

Figure 9, Figure 10, Figure 11, Figure 12, Figure 13 and Figure 14 show the PAR performance of each method under uniform and higher-order distributions, respectively. From Figure 8, under the uniform distribution, for sample sizes of 30, 50, and 100, seq-GNPED’s PAR consistently exceeded those of seq-GDINA. When the sample size increased to 300, seq-GNPED’s PAR was slightly higher (by about 1% to 4%) than that of the seq-GDINA model.

Notably, the PAR advantage of seq-GNPED over seq-GDINA remained stable in small sample scenarios. Specifically, under high item quality, for sample sizes of 30, 50, and 100, seq-GNPED’s PAR was higher than seq-GDINA’s by 3%, 3%, and 2%, respectively. Under medium item quality, the corresponding advantages were 6%, 6%, and 3%. Under low item quality, the advantages reached 7%, 7%, and 6%. These results show that the PAR of the seq-GNPED method is less affected by sample size, and its advantage is more evident in small sample scenarios, further indicating the method’s high application value in small-scale classroom educational testing. Results under the higher-order distribution were basically consistent with those under the uniform distribution.

**(2) Results of simulation study 2 with 10% *Q***-**matrix misspecification rate**

Table 4 presents the results of Simulation Study 2 under the condition of 10% *Q*-matrix misspecification rate, comparing the Pattern Accuracy Ratio (PAR) between the seq-GNPED and seq-GDINA methods across various sample sizes, item quality levels (slipping parameters), and ability distributions.

**Figure 15:**
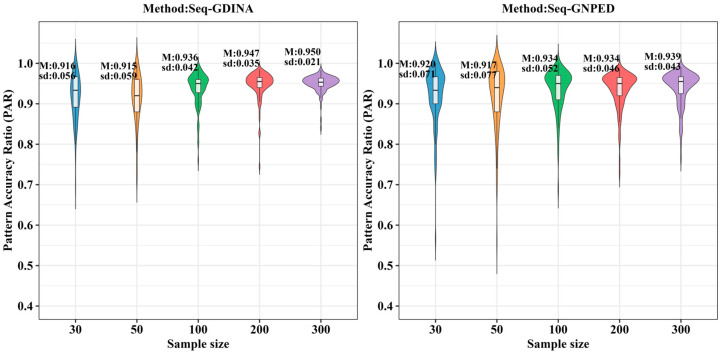
PAR of each method under the condition of uniform distribution with slipping probability 0.05 (10% *Q*-matrix misspecification rate).

**Figure 16:**
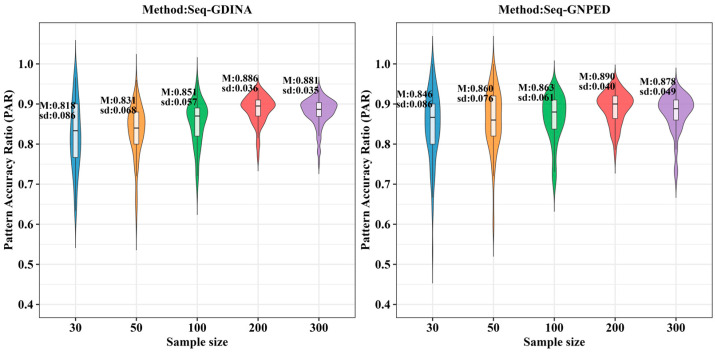
PAR of each method under the condition of uniform distribution with slipping probability 0.1 (10% *Q*-matrix misspecification rate).

**Figure 17:**
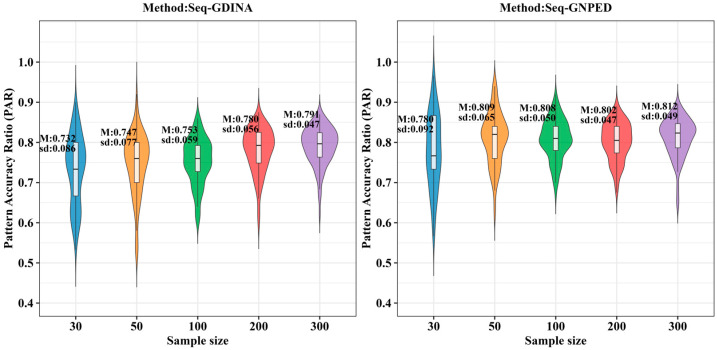
PAR of each method under the condition of uniform distribution with slipping probability 0.15 (10% *Q*-matrix misspecification rate).

**Figure 18:**
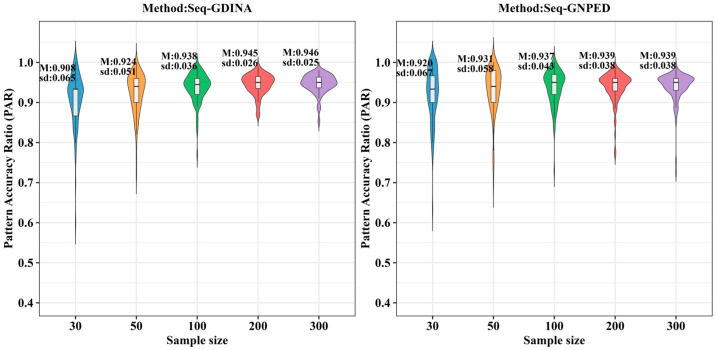
PAR of each method under the condition of higher-order distribution with slipping probability 0.05 (10% *Q*-matrix misspecification rate).

**Figure 19:**
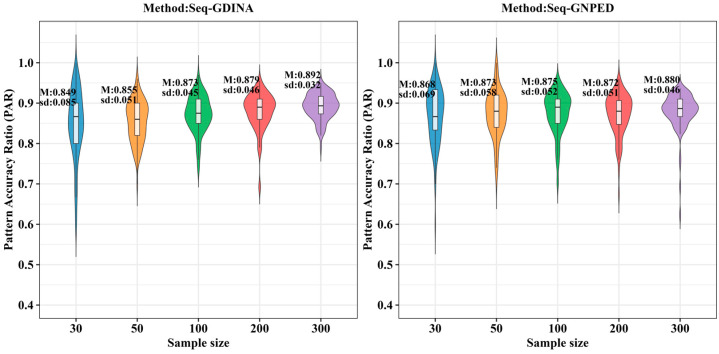
PAR of each method under the condition of higher-order distribution with slipping probability 0.1 (10% *Q*-matrix misspecification rate).

**Figure 20:**
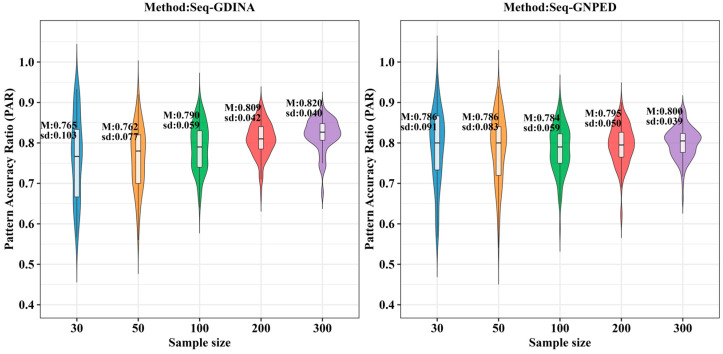
PAR of each method under the condition of higher-order distribution with slipping probability 0.15 (10% *Q*-matrix misspecification rate).

Figure 15, Figure 16, Figure 17, Figure 18, Figure 19 and Figure 20 show that the relative performance of the two methods varied systematically with sample size, item quality, and distribution type. When item quality was low to moderate (s = 0.10 or 0.15), seq-GNPED generally outperformed seq-GDINA in most conditions, particularly in small sample scenarios. Specifically, for sample sizes of 30, 50, and 100, seq-GNPED achieved higher PAR than seq-GDINA under both uniform and higher-order distributions. This advantage persisted for N = 200 under the uniform distribution. However, under the higher-order distribution with N = 200 and 300 at the same item quality levels (s = 0.10 or 0.15), seq-GDINA demonstrated slightly superior PAR instead. When item quality was high (s = 0.05), seq-GDINA consistently yielded slightly better PAR than seq-GNPED across sample sizes (200, 300) and both types of ability distributions.

These findings indicate that the seq-GNPED method is particularly advantageous in small sample contexts or lower item quality, while seq-GDINA performs better when item quality is high, especially with larger samples under the higher-order distribution. The pattern suggests that the choice between methods should consider both sample size and item quality in practical applications.

### 4.3. Study 3: Effect of Polytomous Item Proportion on seq-GNPED

#### 4.3.1. Study Design

The experimental factors in this study included: (1) sample size (*N* = 30, 50, 100, 200); (2) item quality (high, medium, low); (3) cognitive diagnosis method (seq-GDINA, seq-GNPED); (4) proportion of polytomously scored items (75%, 50%, 25%); (5) distribution of examinee knowledge states (uniform, higher-order). Each condition was replicated 100 times, and the averages were analyzed.

The proportion of polytomous items was manipulated by adjusting the initial *Q_c_* matrix (Ma & de la Torre, 2016): for 75%, the original matrix was used; for 50%, 5 of the 16 polytomous items were randomly selected and changed to dichotomous response data; and for 25%, 10 items were randomly selected and changed. The conversion rule from polytomous to dichotomous: if an examinee’s score on item *j* equals the item’s maximum score, it is recorded as 1; otherwise as 0. Examinee knowledge states were generated from a uniform distribution, and item parameter settings were the same as in Study 1. The simulation used the seq-GDINA model to generate data, and the two methods were used for analysis.

#### 4.3.2. Results

Table 5 presents the results of Simulation Study 3 under the condition of perfect *Q*-matrix, comparing the Pattern Accuracy Ratio (PAR) between the seq-GNPED and seq-GDINA methods across various sample sizes, item quality levels (slipping parameters), proportion of polytomously scored items, and ability distributions.

Table 6 shows the PAR of each method under different polytomous item proportions. Overall, for sample sizes of 30, 50, 100, and 200, seq-GNPED’s PAR was consistently higher than those of the seq-GDINA methods.

As the proportion of polytomous items decreased, the PAR of all models showed a declining trend, but the decline for seq-GNPED was the smallest. For example, in uniform distribution, under small-sample (*N* = 30), high-quality item conditions, each 25% reduction in polytomous proportion caused seq-GNPED’s PAR to drop only about 2% to 6%, while seq-GDINA’s PAR could drop 8% to 20%. Moreover, in small sample scenarios (*N* = 30/50/100) with only 25% polytomous items, seq-GNPED’s PAR was 10% to 25% higher than seq-GDINA. These results indicate that the pattern classification accuracy of the seq-GNPED method is less affected by the proportion of polytomous items. When the proportion of polytomous items is low, especially when item quality is poor, seq-GNPED’s advantage becomes more significant, demonstrating its potential to maintain robust diagnostic performance even when test information is limited. Results under the higher-order distribution (Table 7) were basically consistent with those under the uniform distribution.

### 4.4. Study 4: Empirical Data of TIMSS 2011

#### 4.4.1. Study Design

The data for this study came from the TIMSS 2011 mathematics assessment, involving 748 U.S. students. The study used the *Q* matrix constructed by Park et al. (2017). This study analyzed 8 items (7 dichotomous, 1 polytomous); their *Q* matrix is shown in Table 8.

To evaluate the performance of seq-GNPED and seq-GDINA on real data, following C.-Y. Chiu et al. (2018), the classification results from the full sample (748 persons) were used as the comparative benchmarks. Two comparison benchmarks had established: full-data seq-GDINA classification results and full-data seq-GNPED classification results. By randomly drawing different numbers of students from the 748 without replacement, test subsamples of different sizes (*N* = 30, 50, 100) were constructed.

Experimental factors included: (1) random sample size; (2) cognitive diagnosis model (seq-GDINA, seq-GNPED); and (3) comparison benchmark (seq-GDINA, seq-GNPED). Each condition was replicated 100 times to reduce random error. In addition to PAR, the evaluation metric included the Attribute Accuracy Rate (AAR), calculated as:

(25) $$\mathrm{AAR}\;=\frac{1}{N\times K}\sum_{i=1}^{N}\sum_{k=1}^{K}I\left({\hat{\alpha }}_{\mathrm{ik}}\;=\;\alpha_{\mathrm{ik}}\right)$$

where $I\left({\hat{\alpha }}_{\mathrm{ik}}\;=\;\alpha_{\mathrm{ik}}\right)\;=\;1$ if examinee i’s estimate ${\hat{\alpha }}_{\mathrm{ik}}$ on attribute k matches the true value $\alpha_{\mathrm{ik}}$, and 0 otherwise. N is the total number of examinees, and K is the total number of attributes. Higher AAR indicates more accurate attribute-level estimation.

It should be noted that using full sample parametric model classification results as a benchmark (C.-Y. Chiu et al., 2018) implicitly assumes that the parametric model can provide relatively accurate estimates in large samples. Although this assumption is difficult to guarantee in the strict sense, parameter estimation based on large samples is generally more stable and closer to true conditions than small sample estimation, making it reasonably justifiable as a benchmark.

#### 4.4.2. Results

Table 9 shows the PAR and AAR results based on the two different benchmarks. When using the full-data seq-GDINA classification results as the benchmark, under small sample conditions (*N* = 30, 50, 100), the seq-GNPED method’s PAR was significantly higher than that of the seq-GDINA model, by 9.8%, 1.1%, and 3.1%, respectively. Meanwhile, seq-GNPED’s AAR was generally higher than seq-GDINA’s in most small sample conditions.

When using the full-data seq-GNPED classification results as the benchmark, the advantage of the seq-GNPED method was even more pronounced (Table 5). For sample sizes of 30, 50, and 100, its PAR exceeded seq-GDINA’s by 24.5%, 23.0%, and 35.2%, respectively. Furthermore, seq-GNPED’s PAR and AAR values increased steadily with sample size. In summary, regardless of which full-data classification results were used as the benchmark, in small sample scenarios, the seq-GNPED method’s pattern and attribute classification accuracy rates were superior to those of the seq-GDINA model. This further confirms that the seq-GNPED method is more suitable for classroom educational assessment scenarios with limited sample sizes.

### 4.5. Study 5: Empirical Data of Travel Problem-Solving

This study aimed to apply the seq-GNPED method to a localized cognitive diagnostic dataset with a clear external criterion (school quality tier) to systematically examine the method’s validity from the perspectives of internal consistency and external relevance. Validation of internal validity involves examining whether the attribute mastery patterns diagnosed by the seq-GNPED method align with the cognitive attribute hierarchy theory underlying the test design. Validation of external validity focuses on whether the model’s results can effectively reflect known group characteristics related to cognitive ability (i.e., differences between school tiers).

#### 4.5.1. Study Design

**Figure 21:**
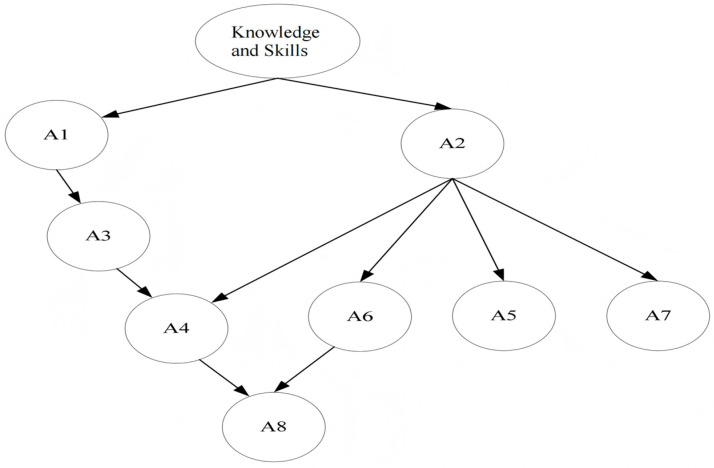
The hierarchy of cognitive attributes of travel problem.

This study utilized the diagnostic test data for elementary school students’ travel problem-solving compiled by C. Kang (2011). The total number of items was 17, including 11 polytomously scored items and 6 dichotomously scored items (Table 10). The sample consisted of 1240 fifth-grade students, who were divided into three tiers based on the external criterion of their school’s teaching quality: “high-performing schools” (*n* = 135), “medium-performing schools” (*n* = 853), and “low-performing schools” (*n* = 252). The test aimed to diagnose eight core cognitive attributes required for solving mathematical travel word problems. Their specific definitions are as follows (Figure 21, C. Kang, 2011). A1: Basic Arithmetic Operations. Refers to the ability to perform addition, subtraction, multiplication, and division calculations (e.g., calculating 58 × 6). A2: Quantitative Relationships in Simple Travel Problems. Refers to the ability to understand and apply the fundamental formula “distance = speed × time” to solve simple travel problems without directional changes (e.g., finding speed given a distance of 180 km and a time of 3 h). A3: Multi-step Operations. Refers to the ability to plan and execute multi-step arithmetic operations involving multiple parentheses or multi-level quantitative relationships (e.g., solving complex problems involving composite expressions like “((600 ÷ 2) − 30) ÷ (5 − 2)”). A4: Quantitative Relationships in Complex Travel Problems. Refers to the ability to analyze problem scenarios (e.g., meeting, parting, pursuit involving directions like opposite, same, or following) and apply corresponding transformed formulas (e.g., involving “sum of speeds” or “difference in speeds”) to solve problems. A5: Identifying Implicit Conditions. Refers to the ability to identify and deduce conditions that are not explicitly stated but are necessary for solving the problem from the problem description (e.g., inferring the actual travel time for Uncle Li based on the statement “Uncle Li took 2 h longer than Uncle Chen”). A6: Relational Representation. Refers to the representational ability to screen key quantitative information from a problem and summarize it into a quantitative relationship that can be directly used for formulating a calculation (e.g., selecting necessary data from multiple pieces of information and formulating the equation “speed = distance/time”). A7: Schematic Representation. Refers to the higher-level representational ability to use visual tools such as line segment diagrams with directional and distance markers to represent and analyze complex quantitative relationships (e.g., using a line diagram to illustrate positional and distance relationships in a two-vehicle meeting problem). A8: Algebraic Nature of Items. Refers to the algebraic thinking ability to recognize unknown quantities in a problem and adopt the strategy of setting up formal algebraic equations to solve it (e.g., setting up an unknown variable and formulating an equation to solve a meeting problem where “A’s speed is 4 times B’s speed”).

The test employed a mixed scoring format. The seq-GNPED method was applied to analyze the response sequences of all students to estimate their attribute mastery patterns.

The core evaluation metric was the Attribute Mastery Rate (AMR), calculated as follows:

(26) $${\mathrm{AMR}}_{k}=\frac{N_{\mathrm{mastery},k}}{N}$$

where $N_{\mathrm{mastery},k}$ represents the number of students classified as having mastered attribute k, and N is the total number of examinees. The AMR intuitively reflects the overall mastery level of a specific group on a given attribute. Internal validity was assessed by analyzing the degree of fit between the overall AMR pattern for all students and theoretical expectations. External validity was verified by examining whether the differential AMR patterns across different school tiers on each attribute were reasonable.

#### 4.5.2. Results

The overall attribute mastery rates diagnosed by the model are presented in Table 11. The results show significant and logical variation in mastery rates across attributes. Attributes A1 (Basic Arithmetic Operations) and A2 (Quantitative Relationships in Simple Travel Problems), representing foundational prerequisite skills, had the highest mastery rates (0.889 and 0.848, respectively). As the cognitive complexity of the attributes increased, mastery rates systematically decreased. Attributes involving advanced representation and abstract thinking, A7 (Schematic Representation) and A8 (Algebraic Nature of Items), showed the lowest mastery rates (0.230 and 0.095, respectively). More importantly, the theoretical prerequisite relationships among attributes were clearly reflected in the data. For instance, the mastery rate of A3 (Multi-step Operations, 0.543), a foundation for handling complex motion relationships in A4, was significantly higher than that of A4 (Quantitative Relationships in Complex Travel Problems, 0.348). This structural pattern, consistent with the rule of cognitive development, provides strong evidence for the internal validity of the seq-GNPED method.

**Figure 22:**
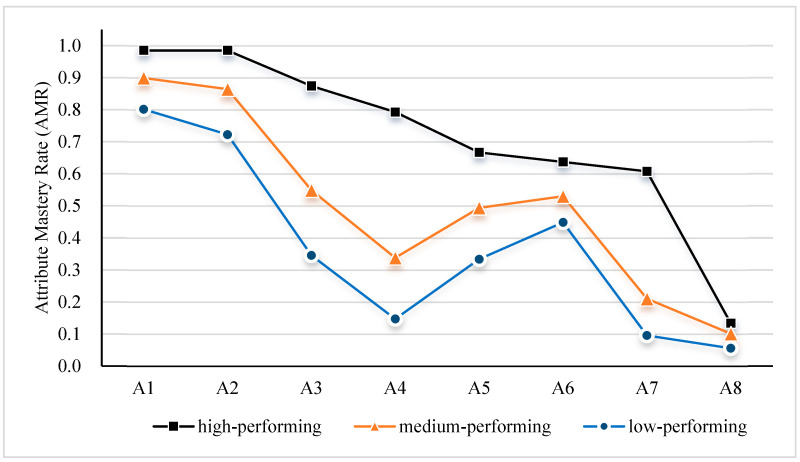
Attribute Mastery Rate (AMR) by school.

The attribute mastery rates by school tier are shown in Figure 22. The data reveal a consistent and stable gradient of “high-performing > medium-performing > low-performing” schools across all eight attributes. This differential pattern highly aligns with the external criterion of school teaching quality, constituting strong support for the seq-GNPED method’s external validity. Notably, the magnitude of differences across attributes of varying difficulty holds practical significance. For basic attributes A1 and A2, the absolute differences between school tiers were smaller. In contrast, for attributes requiring complex cognitive processing (e.g., A3 and A4, which involve multi-step operations and complex relationships, and A7, which requires advanced representation), the gaps in mastery rates between school tiers widened dramatically. For example, for attribute A7 (Schematic Representation), the mastery rate in high-performing schools (0.607) was more than six times that in low-performing schools (0.095). This differential structure accurately reflects the uneven development of higher-order thinking skills among students in different educational environments. For the most advanced attribute, A8 (Algebraic Nature of Items), the mastery rates were extremely low across all school tiers, revealing a common instructional bottleneck for this algebraic thinking skill in primary school education.

In summary, by applying the seq-GNPED method to the travel problem-solving dataset with an external validity criterion, this study confirms that the seq-GNPED’s diagnostic results possess satisfactory internal and external validity. The seq-GNPED can not only output knowledge state patterns consistent with cognitive theory but also sensitively capture and quantify the divergence in group cognitive structures arising from real educational environment differences. This provides a solid validity foundation for its application in practical educational assessment, particularly in serving differentiated instruction and precise intervention.

## 5. Discussion and Conclusions

Addressing the current lack of robust diagnostic methods for polytomously scored data under small sample conditions in cognitive diagnostic assessment, this study proposed a general nonparametric cognitive diagnosis method for polytomous response data—seq-GNPED. By constructing a weighted ideal category response, introducing collapsed attribute classes and an iterative classification mechanism, this method achieves flexible modeling and robust diagnosis of polytomous response data within a nonparametric framework. This chapter systematically summarizes the method’s advantages, explains why it outperforms parametric methods in small samples, and proposes specific directions for future research.

### 5.1. Summary of Method Advantages and Research Implications

The seq-GNPED method demonstrates significant advantages both theoretically and practically, reflected in the following aspects, which in turn bring important implications for educational assessment practice:

**(1) Strong robustness in small samples and immediate support for classroom assessment.** A series of simulation studies showed that under small sample conditions (sample sizes 30 to 100), seq-GNPED’s pattern accuracy rate (PAR) significantly exceeded those of parametric methods seq-DINA and seq-GDINA, especially when item quality was low or the proportion of polytomous items decreased. The reason seq-GNPED possesses this advantage is that it employs the same core algorithm as GNPC. This algorithm determines weights through a data-driven approach and ultimately completes pattern classification via distance metrics (C.-Y. Chiu et al., 2018). This makes it particularly suitable for practical scenarios like classroom formative assessment and regional small-scale testing. The method provides teachers with the possibility of real-time cognitive diagnosis in classrooms of dozens of students, helping to dynamically identify student knowledge states during instruction and implement precise interventions.

**(2) Combining generality and model flexibility.** seq-GNPED inherits the core idea of GNPC, adaptively integrating conjunctive and disjunctive response mechanisms through data-driven weights $w_{\mathrm{ljh}}$, flexibly capturing complex item–attribute relationships. Under different data-generating mechanisms, seq-GNPED consistently showed high diagnostic consistency, indicating good model robustness and broad applicability. This characteristic enables the scientific use of many polytomously scored items such as constructed-response items and performance tasks, avoiding information loss from traditional dichotomization, allowing more detailed assessment of students’ thinking processes and ability development.

**(3) High computational efficiency, easy implementation and promotion, lowering technical application barriers.** Unlike parametric methods that rely on complex iterative estimation algorithms, seq-GNPED is based on algebraic operations and an iterative classification process, offering fast computation and low implementation barriers. This reduces the professional demands placed on users when applying cognitive diagnosis in practice, helping to promote the technology’s transition from research to teaching practice, empowering front-line teachers to conduct diagnostic assessment without strong psychometric backgrounds, and facilitating the implementation of the assessment for learning philosophy.

**(4) The practical value of seq-GNPED is clearly demonstrated through its application in real-world educational settings.** As illustrated in Empirical Study 5, the method serves dual diagnostic purposes that directly support classroom instruction. At the individual level, it generates detailed cognitive profiles for each student, enabling teachers to precisely identify specific attribute weaknesses. For example, a student may demonstrate mastery of basic operations (A1) yet struggle with schematic representation (A7), allowing for targeted instructional interventions tailored to each learner’s needs. This granular diagnostic information empowers teachers to move beyond one-size-fits-all instruction and implement genuinely individualized teaching strategies. At the group level, by aggregating individual diagnostic results to calculate attribute mastery rates, seq-GNPED reveals collective learning patterns across the entire class. In Study 5, this approach showed that foundational skills achieved mastery rates above 85%, while higher-order cognitive attributes fell below 25%, providing teachers with empirical evidence to adjust curriculum focus, allocate instructional time more effectively, and design targeted remediation for commonly problematic attributes. Furthermore, the method’s native support for polytomous items—which better capture students’ partial knowledge and thinking processes than dichotomous scoring—encourages the use of constructed response tasks in classroom assessments, thereby enhancing the diagnostic richness of collected data. Together, these capabilities position seq-GNPED as a practically valuable tool that bridges the gap between theoretical cognitive diagnosis models and everyday classroom instructional decision making.

### 5.2. Why Nonparametric Methods Outperform seq-GDINA in Small Samples

The fundamental reasons why seq-GNPED outperforms parametric method seq-GDINA under small sample conditions can be attributed to the method’s inherent low dependence on sample size and its effective utilization of small sample information through algorithm design. Specific mechanisms are as follows:

**(1) seq-GNPED leverages distance based classification and class collapsing to deliver stable.** Parametric methods like seq-GDINA require estimating numerous item parameters (e.g., intercept, main effects, interaction effects) to establish a response probability model, a process highly sensitive to sample size. Small samples easily lead to large estimation variance, difficult iterative convergence, or local optima. In contrast, seq-GNPED bypasses parameter estimation, directly classifying based on the distance between observed response patterns and ideal patterns. Its objective function (Euclidean distance) is more stable in small samples, less affected by extreme responses or sparse data. Moreover, seq-GNPED reduces the number of potential categories to be distinguished by collapsing attribute classes to the item-category level. For example, in a category examining 3 attributes, collapsing yields only $2^{3}\;=\;8$ classes, far fewer than the $2^{K}$ patterns in the full attribute space. This design aggregates more examinees within each class, increasing local sample density, making weight ${\hat{w}}_{\mathrm{ljh}}$ estimation more stable in small samples and enhancing classification reliability.

**(2) seq-GNPED employs a data-adaptive weighting mechanism and an iterative optimization process to achieve robust cognitive diagnosis.** seq-GNPED automatically adjusts the mixture proportion of conjunctive and disjunctive components via data-driven weights, without pre-specifying the item response function form. This flexibility allows adaptation to the true cognitive mechanisms behind different items, avoiding systematic bias due to model misspecification (e.g., incorrect assumption of compensatory or non-compensatory nature). This adaptive ability is particularly important in small samples, where the true mechanism is harder to accurately predict from limited data. Moreover, seq-GNPED employs an iterative classification process, with initial classification based on a unsaturated nonparametric method (e.g., NPC), followed by gradual optimization through weight updates and reclassification. Simulation studies show that even if initial classification contains errors, the iterative process can quickly converge to a more stable solution in small samples, demonstrating good self-correction and robustness. The theoretical advantages outlined above are fully supported by the empirical results of this study. Analysis of the TIMSS 2011 data in Study 4 demonstrates that, in small-scale testing contexts with limited sample sizes, the pattern accuracy rate of seq-GNPED significantly exceeds that of the parametric seq-GDINA model, highlighting its robustness in small sample applications. Study 5, through analysis of the travel problem-solving data, further confirms that the diagnostic results of seq-GNPED are highly consistent with the tiered structure of school teaching quality. Notably, for higher-order cognitive attributes, the method sensitively reflects significant differences in student abilities across diverse educational environments. This not only validates the sound external validity of the method but also demonstrates its unique value in fully utilizing polytomous scoring information to produce diagnostically meaningful conclusions with educational interpretability. In summary, the empirical results strongly support the feasibility and effectiveness of seq-GNPED as a practical diagnostic tool suitable for small-sample, polytomous scoring contexts.

### 5.3. Research Limitations and Future Directions

The seq-GNPED method proposed in this study provides an effective analysis tool for the cognitive diagnosis of polytomous response data under small samples but still has room for expansion. The current method is mainly suitable for polytomous items with clear category sequences; it has not yet fully adapted to more complex scoring formats, nor integrated other process information (e.g., response time), nor been applied in adaptive testing systems. Deepening research in these aspects will help improve the method’s practicality, interpretability, and scope of application.

**First, regarding scoring types**, it should be noted that the simulations in this study used data generated by the seq-DINA or seq-GDINA models, respectively, aiming to cover a spectrum of cognitive processes from purely conjunctive to partially compensatory, thereby validating the compatibility of seq-GNPED as a general nonparametric method with different data-generating mechanisms. As a saturated model, seq-GDINA encompasses various response mechanisms, with seq-DINA being a special case; thus, the two studies together constitute a systematic examination of seq-GNPED’s applicability. However, because seq-GNPED itself is developed based on the sequential category assumption, this study did not employ models violating this assumption for data generation. Future research could explore extensions of the seq-GNPED framework that are not constrained by the sequential category assumption to address more complex testing scenarios.

**Second, regarding attribute structure**, incorporating intrinsic knowledge hierarchy relationships within subjects can significantly enhance the pedagogical plausibility of diagnostic results (Huo & de la Torre, 2020). Many learning contents have sequential dependencies, e.g., mastering “addition” is a prerequisite for learning “multiplication.” The current method assumes attribute independence, potentially leading to diagnostic conclusions inconsistent with cognitive rule. Future research could incorporate hierarchical constraints during diagnosis, e.g., when generating candidate attribute patterns, filter only patterns that comply with logically defined knowledge structures; or perform consistency adjustments during iterative classification to ensure the final mastery state aligns with learning paths (Leighton et al., 2004; J. Templin & Bradshaw, 2014). This approach not only helps improve classification stability and efficiency in small samples but also enhances the educational interpretability of results, facilitating teacher understanding and use.

**Third, regarding information fusion**. In parametric models, reaction time data significantly enhances the robustness and precision of item parameter estimation (H. A. Kang et al., 2020; Klein Entink et al., 2009; van der Linden et al., 2010). Introducing process data like response time hopefully enhances the robustness of nonparametric methods in small samples. Response time can reflect examinees’ answering speed, familiarity, and cognitive load, providing auxiliary information for judging their knowledge state. For example, a comprehensive distance metric combining score and response time could be constructed, so classification considers both “whether correct” and “answering speed.” In small sample scenarios, such multi-dimensional information fusion can reduce misclassification due to data sparsity, improving diagnostic stability and reliability. This method is relatively straightforward technically, and response time data is easily obtained in computerized testing, offering good application prospects.

**Fourth, regarding missing data.** A practical issue not addressed in the current study is the handling of missing data. In real classroom assessments, it is common for students to skip items or leave partial responses incomplete. Several strategies could be employed to handle missing data in seq-GNPED: (1) use complete datasets to estimate weight parameters, then fix these weights for the classification of all students (including those with missing data); (2) in classroom testing contexts assessing cognitive abilities, non-response can typically be regarded as indicating lack of ability, so missing responses could be scored as 0; or (3) appropriate missing data imputation methods (e.g., mean imputation, nearest neighbor imputation) could be applied to impute missing values before analysis. Future research should systematically investigate the effects of different missing data mechanisms (e.g., MCAR, MAR) and missing rates on seq-GNPED’s classification accuracy, and develop missing data handling methods specifically designed for the nonparametric distance-based framework to enhance the method’s practical applicability.

**Finally, regarding system application**. Cognitive diagnostic computerized adaptive testing (CD-CAT) integrates the refined analytical capabilities of cognitive diagnostic models (CDMs) with the dynamic adaptive advantages of computerized adaptive testing (CAT) (Cheng, 2009), aiming to efficiently and in real time diagnose examinees multidimensional knowledge states through adaptive item selection mechanisms (P. Chen et al., 2012; P. Chen & Wang, 2015; Lin & Chang, 2019; Liu et al., 2013). Extending seq-GNPED to cognitive diagnostic computerized adaptive testing (CD-CAT) has important practical value. This requires exploring several key issues, e.g., how to design adaptive item selection strategies within a nonparametric framework to quickly narrow candidate attribute patterns; how to achieve online dynamic estimation of weights for new items; and how to formulate flexible test termination rules based on diagnostic confidence. This work could leverage seq-GNPED’s small sample friendly characteristics to significantly improve test efficiency while ensuring diagnostic accuracy.

Looking ahead, seq-GNPED could be further expanded in the following directions. First, conduct more extensive real-world classroom application studies, collecting feedback from frontline teachers to validate the method’s feasibility and acceptability in teaching assessment. Second, explore integrating seq-GNPED with computerized adaptive testing (CAT) to develop nonparametric adaptive diagnostic systems suitable for small sample contexts, further enhancing testing efficiency. Third, as mentioned above, investigate the method’s performance and coping strategies under complex conditions such as *Q*-matrix misspecification, missing data, and attribute hierarchies. Finally, attempt to extend the core ideas of seq-GNPED to broader item types and more diverse data types (such as response times and process data), constructing a more comprehensive nonparametric cognitive diagnostic analysis framework.

## Figures and Tables

**Table 1:** *Q_c_*-matrix for study 1, study 2 and study 3.

Item	Cat	A1	A2	A3	A4	A5
1	1	1	0	0	0	0
1	2	0	1	0	0	0
2	1	0	0	1	0	0
2	2	0	0	0	1	0
3	1	0	0	0	0	1
3	2	1	0	0	0	0
4	1	0	0	0	0	1
4	2	0	0	0	1	0
5	1	0	0	1	0	0
5	2	0	1	0	0	0
6	1	1	0	0	0	0
6	2	0	1	1	0	0
7	1	0	0	1	0	0
7	2	0	0	0	1	1
8	1	0	0	0	0	1
8	2	1	1	0	0	0
9	1	0	0	0	1	1
9	2	0	0	1	0	0
10	1	0	1	0	1	0
10	2	1	0	0	0	0
11	1	1	1	0	0	0
11	2	0	0	0	0	1
12	1	1	1	1	0	0
12	2	0	0	0	1	1
13	1	1	1	0	0	0
13	2	0	0	1	1	1
14	1	1	0	1	0	0
14	2	0	0	0	1	0
14	3	0	0	0	0	1
15	1	0	0	0	0	1
15	2	0	0	1	1	0
15	3	0	1	0	0	0
16	1	1	0	0	0	0
16	2	0	1	0	0	0
16	3	0	0	1	1	0
17	1	1	0	0	0	0
18	1	0	1	0	0	0
19	1	0	0	1	0	0
20	1	0	0	0	1	0
21	1	0	0	0	0	1

**Table 2:** Summary of factors and results in simulation study 1.

Factor	Level	Pattern Accuracy Ratio (PAR)
sample size (*N*)	30, 50, 100, 200, 300	seq-GNPED > seq-GDINA
item quality (*s*)	0.05, 0.10, 0.15	seq-GNPED > seq-GDINA
distribution	uniform, higher-order	seq-GNPED > seq-GDINA

**Table 3:** Summary of factors and results in simulation study 2 (0% *Q*-matrix misspecification rate).

Factor	Level	Pattern Accuracy Ratio (PAR)
sample size (*N*)	30, 50, 100, 200, 300	seq-GNPED > seq-GDINA
item quality (*s*)	0.05, 0.10, 0.15	seq-GNPED > seq-GDINA
distribution	uniform, higher-order	seq-GNPED > seq-GDINA

**Table 4:** Results in simulation study 2 (10% *Q*-matrix misspecification rate).

Sample Size (*N*)	Item Quality (*s*)	Distribution	Pattern Accuracy Ratio (PAR)
30, 50	0.05, 0.10, 0.15	uniform, higher-order	seq-GNPED > seq-GDINA
100	0.10, 0.15	uniform, higher-order	seq-GNPED > seq-GDINA
200	0.10, 0.15	uniform	seq-GNPED > seq-GDINA
100, 200, 300	0.05	uniform, higher-order	seq-GDINA > seq-GNPED
200, 300	0.10, 0.15	higher-order	seq-GDINA > seq-GNPED

**Table 5:** Summary of factors and results in simulation study 3.

Factor	Level	Pattern Accuracy Ratio (PAR)
sample size (*N*)	30, 50, 100, 200	seq-GNPED > seq-GDINA
proportion of polytomous items	75%, 50%, 25%	seq-GNPED > seq-GDINA
item quality (*s*)	0.05, 0.10, 0.15	seq-GNPED > seq-GDINA
distribution	uniform, higher-order	seq-GNPED > seq-GDINA

**Table 6:** PAR of each method under different proportions of polytomous scoring items (uniform).

Proportion	Item Quality	Sample Size	seq-GDINA		seq-GNPED	
			Mean	Sd	Mean	Sd
75%	high	30	0.928	0.057	**0.964**	0.036
		50	0.938	0.041	**0.966**	0.025
		100	0.955	0.025	**0.965**	0.019
		200	0.961	0.016	**0.967**	0.015
	medium	30	0.852	0.078	**0.911**	0.054
		50	0.864	0.063	**0.921**	0.037
		100	0.885	0.037	**0.923**	0.028
		200	0.898	0.028	**0.921**	0.021
	low	30	0.769	0.091	**0.840**	0.063
		50	0.767	0.069	**0.845**	0.058
		100	0.792	0.052	**0.854**	0.040
		200	0.813	0.035	**0.858**	0.026
50%	high	30	0.853	0.084	**0.947**	0.040
		50	0.873	0.075	**0.943**	0.031
		100	0.923	0.038	**0.949**	0.022
		200	0.943	0.020	**0.953**	0.018
	medium	30	0.734	0.108	**0.871**	0.059
		50	0.770	0.088	**0.863**	0.052
		100	0.818	0.059	**0.885**	0.035
		200	0.858	0.032	**0.891**	0.025
	low	30	0.614	0.118	**0.776**	0.074
		50	0.660	0.095	**0.789**	0.070
		100	0.694	0.068	**0.789**	0.042
		200	0.755	0.043	**0.806**	0.030
25%	high	30	0.580	0.152	**0.883**	0.059
		50	0.710	0.123	**0.910**	0.040
		100	0.831	0.099	**0.914**	0.029
		200	0.893	0.056	**0.921**	0.021
	medium	30	0.479	0.125	**0.790**	0.079
		50	0.558	0.111	**0.801**	0.064
		100	0.674	0.099	**0.808**	0.037
		200	0.770	0.050	**0.821**	0.027
	low	30	0.393	0.125	**0.663**	0.095
		50	0.421	0.107	**0.678**	0.064
		100	0.529	0.087	**0.710**	0.045
		200	0.616	0.060	**0.700**	0.038

Note: The numbers in bold refer to the best results.

**Table 7:** PAR of each method under different proportions of polytomous scoring items (higher-order).

Proportion	Item Quality	Sample Size	seq-GDINA		seq-GNPED	
			Mean	Sd	Mean	Sd
75%	high	30	0.949	0.048	**0.965**	0.039
		50	0.949	0.035	**0.967**	0.025
		100	0.958	0.024	**0.967**	0.020
		200	0.961	0.014	**0.966**	0.014
	medium	30	0.875	0.064	**0.912**	0.052
		50	0.886	0.055	**0.904**	0.040
		100	0.890	0.034	**0.907**	0.032
		200	0.903	0.024	**0.911**	0.024
	low	30	0.796	0.089	**0.838**	0.068
		50	0.786	0.072	**0.826**	0.061
		100	0.817	0.050	**0.835**	0.037
		200	0.838	0.037	**0.843**	0.030
50%	high	30	0.869	0.084	**0.945**	0.043
		50	0.908	0.050	**0.939**	0.033
		100	0.929	0.037	**0.948**	0.025
		200	0.950	0.020	**0.953**	0.016
	medium	30	0.769	0.090	**0.860**	0.067
		50	0.793	0.089	**0.865**	0.048
		100	0.843	0.046	**0.872**	0.040
		200	0.870	0.036	**0.875**	0.031
	low	30	0.655	0.107	**0.759**	0.081
		50	0.676	0.092	**0.749**	0.061
		100	0.742	0.067	**0.779**	0.042
		200	0.779	0.044	**0.783**	0.031
25%	high	30	0.666	0.133	**0.891**	0.062
		50	0.723	0.128	**0.907**	0.047
		100	0.814	0.122	**0.911**	0.033
		200	0.875	0.089	**0.917**	0.022
	medium	30	0.503	0.130	**0.776**	0.081
		50	0.567	0.108	**0.796**	0.059
		100	0.686	0.101	**0.800**	0.042
		200	0.744	0.103	**0.809**	0.034
	low	30	0.438	0.130	**0.651**	0.098
		50	0.464	0.114	**0.672**	0.073
		100	0.535	0.089	**0.679**	0.047
		200	0.621	0.092	**0.682**	0.039

Note: The numbers in bold refer to the best results.

**Table 8:** *Q* matrix for real data of TIMSS 2011.

Item	TIMSS Item ID	Cat	A1	A2	A3	A4	A5
1	M042198C	1	1	0	0	0	0
2	M042235	1	0	1	0	0	0
3	M042150	1	0	0	1	0	0
4	M042300Z	1	0	0	0	1	1
4	M042300Z	2	0	0	1	0	0
5	M042169A	1	0	0	0	0	1
6	M032295	1	0	1	0	0	0
7	M032331	1	0	0	1	1	0
8	M032398	1	0	0	1	0	0

Notes. A1, patterns; A2, expressions, equations and functions; A3, lines, angles and shapes; A4, location and movement; and A5, data organization, representation and interpretations.

**Table 9:** Classification accuracy of each method in real data.

Sample Size	Comparative Benchmarks	PAR		AAR	
		seq-GDINA	seq-GNPED	seq-GDINA	seq-GNPED
30	seq-GDINA	0.442	0.540	0.872	0.877
	seq-GNPED	0.562	0.807	0.892	0.956
50	seq-GDINA	0.451	0.566	0.868	0.885
	seq-GNPED	0.581	0.811	0.899	0.958
100	seq-GDINA	0.551	0.582	0.894	0.889
	seq-GNPED	0.486	0.838	0.873	0.962

**Table 10:** *Q* matrix for real data of travel problem-solving.

Item	Cat	A1	A2	A3	A4	A5	A6	A7	A8
1	1	1	0	0	0	0	0	0	0
2	1	1	0	0	0	0	0	0	0
2	2	0	0	1	0	0	0	0	0
3	1	0	1	0	0	0	0	0	0
4	1	0	1	0	0	0	0	0	0
4	2	0	0	0	0	0	0	1	0
5	1	1	1	0	0	0	0	0	0
6	1	1	1	1	0	1	0	0	0
7	1	0	1	0	0	1	0	0	0
8	1	0	1	0	0	0	0	0	0
8	2	0	0	0	0	0	1	0	0
9	1	0	1	0	0	1	0	0	0
9	2	0	0	0	0	0	1	1	0
10	1	1	1	1	1	0	0	0	0
10	2	0	0	0	0	0	1	0	0
10	3	0	0	0	0	0	0	0	1
11	1	1	1	0	0	0	0	0	0
11	2	0	0	0	1	0	1	1	0
11	3	0	0	1	0	0	0	0	0
12	1	1	1	0	0	0	0	0	0
12	2	0	0	1	1	0	1	1	0
12	3	0	0	0	0	1	0	0	0
13	1	1	1	0	0	0	0	0	0
13	2	0	0	0	1	0	0	0	0
13	3	0	0	1	0	0	0	0	0
14	1	1	1	1	1	0	0	1	0
15	1	1	1	0	0	0	0	0	0
15	2	0	0	1	1	1	1	1	0
15	3	0	0	0	0	0	0	0	1
16	1	1	1	0	0	0	0	0	0
16	2	0	0	0	0	1	0	0	0
17	1	1	1	0	0	0	0	0	0
17	2	0	0	0	0	1	0	0	0
17	3	0	0	1	1	0	0	0	0
17	4	0	0	0	0	0	0	1	0

**Table 11:** Overall Attribute Mastery Rate (AMR) based on seq-GNPED.

A1	A2	A3	A4	A5	A6	A7	A8
0.889	0.848	0.543	0.348	0.480	0.525	0.230	0.095

## Data Availability

The research data and materials are available on https://osf.io/rfm3c/, accessed after 1 April 2026.

## Supplementary Materials

The supporting information can be downloaded at: https://osf.io/rfm3c/, accessed after 1 April 2026.

## Abbreviations

The following abbreviations are used in this manuscript:

GNPC	Generalized nonparametric classification (GNPC) method
seq-GNPED	Sequential generalized nonparametric classification method with Euclidean distance
